# Experience-dependent resonance in amygdalo-cortical circuits supports fear memory retrieval following extinction

**DOI:** 10.1038/s41467-020-18199-w

**Published:** 2020-08-31

**Authors:** Minagi Ozawa, Patrick Davis, Jianguang Ni, Jamie Maguire, Thomas Papouin, Leon Reijmers

**Affiliations:** 1 Department of Neuroscience, School of Medicine, Tufts University, Boston, MA USA; 2 Graduate Program in Neuroscience, Graduate School of Biomedical Sciences, Tufts University, Boston, MA USA; 3 Medical Scientist Training Program, Graduate School of Biomedical Sciences, Tufts University, Boston, MA USA; 4 Department of Neuroscience, Washington University in St. Louis, School of Medicine, St. Louis, MO USA; 5 Present Address: Boston Combined Residency Program (Child Neurology), Boston Children’s Hospital, Boston, MA USA

**Keywords:** Learning and memory, Neural circuits

## Abstract

Learned fear and safety are associated with distinct oscillatory states in the basolateral amygdala (BLA) and medial prefrontal cortex (mPFC). To determine if and how these network states support the retrieval of competing memories, we mimicked endogenous oscillatory activity through optogenetic stimulation of parvalbumin-expressing interneurons in mice during retrieval of contextual fear and extinction memories. We found that exogenously induced 4 Hz and 8 Hz oscillatory activity in the BLA exerts bi-directional control over conditioned freezing behavior in an experience- and context-specific manner, and that these oscillations have an experience-dependent ability to recruit distinct functional neuronal ensembles. At the network level we demonstrate, via simultaneous manipulation of BLA and mPFC, that experience-dependent 4 Hz resonance across BLA-mPFC circuitry supports post-extinction fear memory retrieval. Our findings reveal that post-extinction fear memory retrieval is supported by local and interregional experience-dependent resonance, and suggest novel approaches for interrogation and therapeutic manipulation of acquired fear circuitry.

## Abstract (web summary)

Theta range oscillations in the basolateral amygdala (BLA) and the medial prefrontal cortex (mPFC) are associated with conditioned fear. Here, the authors use exogenous oscillatory stimulation of the BLA and mPFC in mice to determine the dynamic roles of theta-range oscillatory states across conditioned fear and extinction learning.

## Introduction

The mammalian brain is a complex, nonlinear device that operates across many timescales and abstract levels^1^. A central purpose of neuroscience is to elucidate how flexible, yet robust behavioral manifestations arise from this daunting complexity. One proposed solution to this problem lies in the ubiquitous phenomenon of circuit oscillations, which emerge from but also constrain cellular and synaptic behavior, as well as permit rapid and flexible switching between network states^2^. The functional relevance of neural oscillatory phenomena is supported by the diverse and widely observed associations between oscillations and behavior^3^. These behavioral associations may be facilitated in part by oscillatory recruitment of functional neuronal ensembles, such as place-cell firing during hippocampal theta oscillations^4^. Recruitment of neuronal ensembles to specific oscillations is thought to occur in part through subthreshold resonance properties of neurons, which can enable neurons to amplify their response to exogenous oscillatory input at a given frequency^5^. Subthreshold resonance has been shown to translate into spiking resonance in behaving mice, supporting a role for resonance in the processing of information by neural circuits^6^. This raises the intriguing possibility that learning-induced changes in the resonance properties of a neural circuit might contribute to the initial encoding of memories, and the subsequent oscillatory control of their retrieval^7^.

Establishing a possible relationship between oscillatory activity, resonance, and memory function requires a memory paradigm with known circuit components, robust behavioral readouts, and distinct oscillatory activity patterns. One such memory paradigm is that of conditioned fear and extinction learning, which leads to the formation of competing fear and extinction memories with opposing effects on fear behavior^8^. Converging evidence points to circuits connecting the basolateral amygdala (BLA) and the medial prefrontal cortex (mPFC) as key nodes for both the encoding and retrieval of these competing memories^9,10^. Distant brain areas like the BLA and mPFC are theorized to functionally communicate with each other via synchronized neural oscillations that allow for coordinated spiking activity between brain regions^11–13^. Indeed, distinct synchronous oscillations in the theta-range (~3–12 Hz) have been detected in BLA-mPFC circuits, and have been shown to correlate with either increased or decreased experience-dependent fear behavior^14–21^. Interestingly, both BLA and mPFC neurons can display theta-range resonance^22–27^. Theta-range resonance in BLA-mPFC circuits could therefore support the observed association of theta-range oscillations with experience-dependent fear behavior.

In order to study the dynamic roles of theta-range oscillatory states across conditioned fear and extinction learning, we combined exogenous oscillatory stimulation of the BLA and mPFC with local-field potential (LFP) and unit recordings in mice subjected to contextual fear conditioning and extinction. Exogenous optical stimulation was performed at 4 Hz and 8 Hz, thereby mimicking the endogenous theta-range oscillations previously associated with opposing experience-dependent fear and safety states^17,19,20^. By performing identical oscillatory stimulation across multiple time-points throughout the behavioral paradigm, we were able to observe how learning alters the responsivity of the circuitry. We detected experience-dependent changes in the ability of exogenous oscillatory stimulation to alter fear behavior, which were accompanied by parallel experience-dependent changes in the ability of exogenous oscillatory stimulation to (1) synchronize spiking activity locally within the BLA, and (2) recruit interregional interactions between the BLA and mPFC. Thereby, our findings uncover experience-dependent changes in theta-range resonance properties of BLA-mPFC circuits that support the retrieval of fear and extinction memories.

## Results

### 4 Hz and 8 Hz oscillations modulate local BLA spiking

**Fig. 1:**
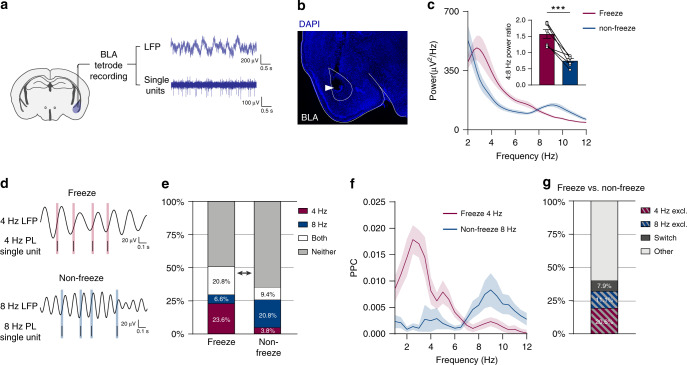
4 Hz and 8 Hz oscillations differentially modulate local BLA spiking activity. **a** Experimental schematic. Thirty two channel tetrode drives were implanted into the BLA to extract both LFP and spiking activity in mice while subjected to fear conditioning and extinction. **b** Example histology image showing tetrode placement after electrolytic lesion. **c** Averaged BLA power spectra during freezing and non-freezing periods used for phase-locking analysis. Inset: quantification as 4:8 Hz power ratio (paired two-tailed *t*-test: *P* = 0.0007, *t*(6) = 6.31, *n* = 7 mice). **d** Example BLA single units that significantly phase-locked (PL) to BLA LFP at 4 Hz during freezing or 8 Hz during non-freezing (significance based on permutation test *P* < 0.05; see Methods section). **e** Comparison of proportion of BLA single units that significantly phase-lock (based on permutation test *P* < 0.05; see Methods section) to 4 Hz only, 8 Hz only, both, or neither during freezing vs. non-freezing behavior (Chi-square test: *X*^2^(3, *n* = 106 units) = 30.12, *P* < 0.0001). **f** Averaged PPC spectra of units that phase-lock to 4 Hz during freezing (*n* = 25 cells) or 8 Hz during non-freezing (*n* = 22 cells). **g** Percentage of BLA single units that exclusively phase-lock to 8 Hz during non-freezing, a distinct subset that exclusively phase-lock to 4 Hz during freezing, and a third group that switches between the two (percentage of all significantly phase-locked units, *n* = 63 units). All error bars and shaded area: mean ± SEM.

**Fig. 2:**
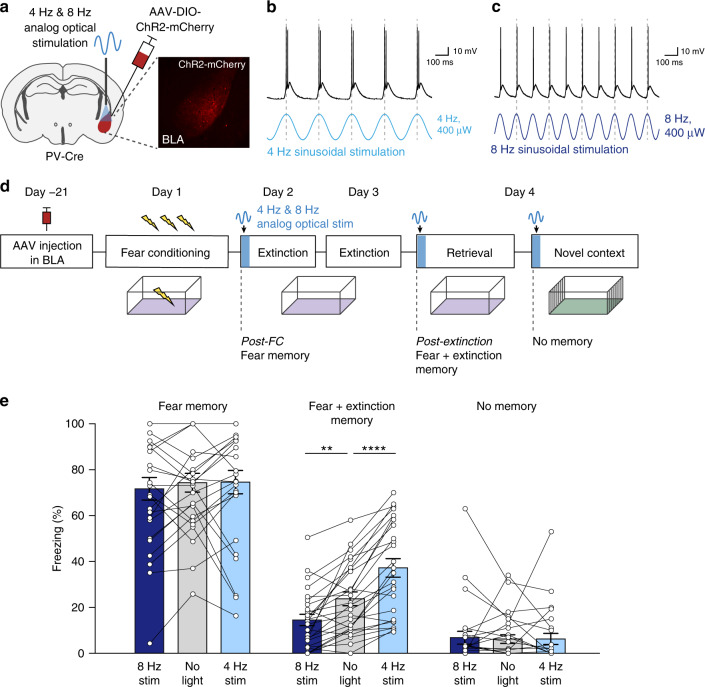
Frequency-based, bidirectional control of post-extinction memory retrieval. **a** Schematic of viral strategy for in vivo optogenetic control of BLA PV-interneurons. **b**, **c** Example traces from acute BLA slices demonstrating effect of sinusoidal optical stimulation on action potential generation in PV-interneurons (also see Supplementary Fig. 2). **d** Schematic of experimental design for standard extinction group (see Supplementary Fig. 3a for experimental design for delayed extinction group; see Methods section for details). **e** Optical stimulation of BLA PV-interneurons has a bidirectional effect on memory retrieval in the conditioned context following, but not before, extinction learning (two-way repeated measures ANOVA: trial *F*(2,50) = 134.4, *P* < 0.0001, stimulation *F*(2,50) = 18.49, *P* < 0.0001, trial × stimulation *F*(4, 100) = 10.15, *P* < 0.0001, *n* = 26 mice; Holm–Sidak’s multiple comparisons test; fear memory: no light vs. 8 Hz stim *t*(100) = 0.94, *P* = 0.57; no light vs 4 Hz stim *t*(100) = 0.09, *P* = 0.93; fear + ext memory: no light vs 8 Hz stim *t*(100) = 3.27, *P* = 0.0015; no light vs 4 Hz stim *t*(100) = 4.8, *P* < 0.0001; no memory: no light vs 8 Hz stim *t*(100) = 0.21, *P* = 0.97; no light vs 4 Hz stim *t*(100) = 0.018, *P* = 0.99). Error bars: mean ± SEM.

We previously identified two competing oscillatory states in the BLA with opposing behavioral associations: a 3–6-Hz (hereafter referred to as 4 Hz) oscillation associated with freezing behavior and a distinct 6–12-Hz (hereafter referred to as 8 Hz) oscillation associated with non-freezing or safety behavior^20^. Since we detected these oscillations in the LFP signal, which reflects the activity of large populations of neurons and is susceptible to volume conduction, the precise relevance of the 4-Hz and 8-Hz oscillations to local BLA microcircuit states remained to be elucidated. We therefore decided to simultaneously record LFPs and spiking activity of units within the BLA of mice subjected to contextual fear conditioning and extinction learning (Figs. 1a–b and 2d), using the same behavioral paradigm as in our previous study^20^. We found, consistent with our previous results, that periods of freezing were associated with a 4-Hz LFP oscillation, whereas periods of non-freezing were associated with an 8 Hz LFP oscillation (Fig. 1c). Next, we compared spike-field phase-locking of units during freezing periods to non-freezing periods (Fig. 1d and Supplementary Fig. 1a–f). For this comparison we analyzed an extinction trial during which mice alternated frequently between freezing and non-freezing behavior in order to follow the activity of the same units during both behaviors. We found single units that significantly phase-lock to local 8 Hz oscillations (20.8% of BLA single-units in non-freeze periods), as well as units that significantly phase-lock to 4 Hz oscillations (23.6% in freezing periods; Fig. 1e–f). We further found that the proportion of BLA single-units that phase-lock to each oscillation shifts as the mouse switches between freezing and non-freezing behavior (Fig. 1e–f). Interestingly, when analyzing phase-locking of units across different behavioral states, we found that some units were capable of switching their phase-locking between the two oscillations. However, a larger proportion of units phase-locked exclusively to 4 Hz during freezing or 8 Hz during non-freezing, suggesting that the two opposing oscillatory states recruit distinct populations of BLA neurons (Fig. 1g).

### Frequency-specific bidirectional control of memory retrieval

The correlational data in Fig. 1 suggest functional roles for 4 Hz and 8 Hz oscillations in organizing BLA neural activity during opposing experience-dependent fear and safety states. We therefore hypothesized that these two oscillatory states support the regulation of fear and extinction memory retrieval. To test this hypothesis, we exogenously induced these two oscillations across varying fear and extinction memory states. We adopted an optogenetic strategy to rhythmically stimulate parvalbumin (PV)-positive interneurons in the BLA because: (1) we previously found that chemogenetic manipulation of PV-interneurons altered the LFP signatures in the BLA during fear and extinction memory retrieval^20^, and (2) previous studies found optogenetic manipulation of PV-interneurons efficient at inducing oscillatory activity in other brain regions^6,19^. We injected a Cre-dependent channelrhodopsin-expressing virus (AAV-DIO-ChR2-mCherry) into the BLA of *PV-Cre* mice, thereby expressing ChR2 exclusively in PV-interneurons within the BLA (Fig. 2a and Supplementary Fig. 4a). Application of a sinusoidal waveform of light at 4 Hz or 8 Hz in acute BLA slices produced rhythmic firing of PV-interneurons aligned to the peaks of the stimulus waveform without changing overall firing rate (Fig. 2b–c and Supplementary Fig. 2), indicating that any differential effects of 4 Hz versus 8 Hz stimulation are due to the temporal pattern, not amount, of PV-interneuron activity.

Mice expressing ChR2 in BLA PV-interneurons were subjected to a behavioral paradigm consisting of contextual fear conditioning followed by extinction learning. We applied sinusoidal optical stimulation at 4 Hz or 8 Hz during three different test trials: a post-fear conditioning trial (fear memory state), a post-extinction learning trial (fear + extinction memory state), and a trial in an unconditioned neutral context (no memory state) (Fig. 2d). This enabled us to examine the effects of identical, rhythmic stimulation across varying memory states, thereby probing the role of network oscillations in memory retrieval per se, rather than simply behavioral control. To first demonstrate that our stimulation protocol led to the intended effect on circuit activity, we recorded BLA LFPs during optogenetic 4 Hz and 8 Hz stimulation. Indeed, we found that 4 Hz and 8 Hz stimulation shifted the BLA power spectrum toward 4 Hz and 8 Hz, respectively (Supplementary Fig. 4c, d). Strikingly, while freezing behavior was not changed by 4 Hz and 8 Hz BLA stimulation in the post-fear conditioning trial, in the subsequent stimulation trial after extinction learning, the same stimulations had opposite effects on fear behavior: specifically, 4 Hz stimulation augmented, and 8 Hz stimulation suppressed freezing behavior (Fig. 2e). When tested in an unconditioned context, 4 Hz and 8 Hz stimulation had no effect (Fig. 2e). Furthermore, these behavioral effects were not merely due to the passage of time, differences in baseline freezing levels, or other non-specific effects (Supplementary fig. 3). Our data demonstrate that exogenous induction of 4 Hz and 8 Hz oscillations in the BLA induces an experience- and context-dependent bidirectional effect on freezing behavior, demonstrating selective effects on the retrieval of a contextual fear and extinction memory.

### 4 Hz and 8 Hz BLA stimulation recruit distinct BLA ensembles

**Fig. 3:**
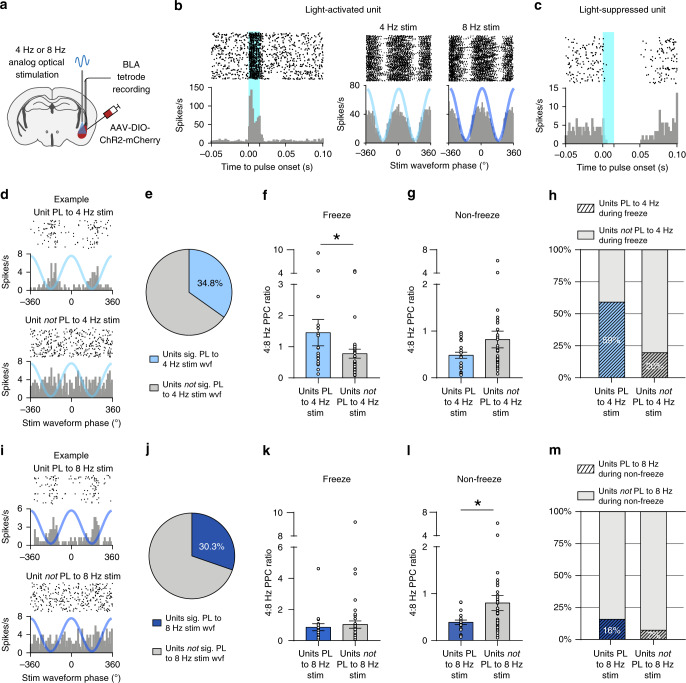
4 Hz and 8 Hz stimulation recruit functionally distinct neuronal ensembles. **a** Experimental schematic. *PV-cre* mice were injected with AAV-DIO-ChR2-mCherry and implanted with optic cannulae and 32 channel tetrode drives in the BLA to extract both LFP and spiking activity in mice subjected to fear conditioning and extinction. **b** Raster plot (top) and spike histogram (bottom) of an example light-activated BLA single-unit, aligned to onset of 15 ms light pulses (left) or to the phases of the 4 Hz and 8 Hz sinusoidal light waveform (right). **c** Same as **b** but for a light-suppressed BLA single-unit. **d** Spike histograms of an example neuron that significantly phase-locks to 4 Hz stimulus waveform (top) and another unit that does not (bottom). **e** Percentage of units that significantly phase-lock (PL) to 4 Hz stimulus waveform during extinction memory retrieval (significance based on permutation test *P* < 0.05, *n* = 23/66 units). **f** Units that significantly phase-lock to 4 Hz stimulation show a higher 4:8 Hz PPC ratio during freezing than units that do not phase-lock to 4 Hz stimulation. (Mann–Whitney test, two-tailed: *U* = 308, *P* = 0.039, *n* = 22 and 41 units). **g** Same as in **f** but during non-freeze periods. Units that significantly phase-lock to 4 Hz stimulation and those that do not, show no difference in 4:8 Hz PPC ratio during non-freezing (Mann–Whitney test, two-tailed: *U* = 326, *P* = 0.25, *n* = 21 and 38 units). **h** Units that are recruited to endogenous freezing-associated 4 Hz oscillations are more likely to phase-lock to the 4-Hz stimulus waveform (Chi-square test: *X*^2^ = 10.09, *P* = 0.0015). **i** Same as **d** but for 8 Hz stimulation. **j** Same as **e** but for 8 Hz stimulation (significance based on permutation test *P* < 0.05, *n* = 20/66 units). **k**, **l** Same as (f-g) but for 8 Hz stimulation. Units that were significantly phase-locked to 8 Hz stimulus waveform had lower 4:8 Hz PPC ratios during non-freezing periods. (Mann–Whitney test, two-tailed: Freeze (k): *U* = 413, *P* = 0.84, *n* = 19 and 45 units; Non-freeze (l): *U* = 256, *P* = 0.026, *n* = 19 and 42 units). **m** Comparison of percentage of units that are recruited to endogenous non-freezing-associated 8 Hz oscillations among units that significantly phase-lock to 8 Hz stimulation vs. those that do not (Chi-square test: *X*^2^ = 1.10, *P* = 0.29). All error bars: mean ± SEM.

To further characterize the role of 4 Hz and 8 Hz activity within BLA, and to elucidate the mechanism by which rhythmic PV-interneuron stimulation can generate experience-dependent behavioral control, we recorded spike data and extracted single-unit activity from the BLA of mice subjected to our conditioning and optogenetic stimulation paradigm (Figs. 2d and 3a). As expected, we found examples of both light-activated and light-suppressed units, consistent with optogenetic depolarization of PV interneurons leading to a fast increase in synaptic inhibition of other neurons (Fig. 3b–c). Interestingly, we also observed evidence of inhibition-induced rebound spiking in BLA, consistent with prior in vitro evidence of PV-interneurons coordinating ensemble activity via inhibition and hyper-polarization induced spiking (Supplementary Fig. 1h–k)^25,27^. Spiking activity of light-activated units was faithfully modulated according to the phase of a sinusoidal light stimulus, with firing rate highest at the peak of the sinusoid (Fig. 3b). Interestingly, the responses of other neurons were more varied in their degree of entrainment to the stimulus waveform (Fig. 3d, i). One possible explanation for the differential behavioral effect of 4 Hz and 8 Hz stimulation is that they recruit different functional ensembles within the BLA. To test for this possibility, we further classified the units according to their capacity to be entrained and recruited to each of the exogenous stimulus waveforms. We found that 34.8% and 30.3% of units significantly phase-locked to the 4 Hz and 8 Hz stimulus waveforms, respectively, in the conditioned context (Fig. 3e, j). We also found units that phase-locked to both waveforms (Supplementary Fig. 1l). Interestingly, the populations of neurons recruited to each stimulation frequency appeared to have distinct properties during endogenous oscillatory activity. Units that were phase-locked to the 4-Hz stimulus waveform were more likely to also be recruited to endogenous, freezing-associated 4 Hz oscillations (Fig. 3h). Furthermore, the phase-locking of these units was shifted toward 4 Hz during freezing, but not non-freezing periods (Fig. 3f–g). Conversely, phase-locking of units recruited to the exogenous 8 Hz stimulation was significantly shifted toward 8 Hz during non-freezing periods (Fig. 3k–m). Taken together, these data reveal that the exogenous stimulus response properties of individual units predict their recruitment to endogenous freezing (4 Hz) or non-freezing (8 Hz) associated oscillations. Interestingly, this suggests that the differential behavioral effects of 4 Hz and 8 Hz BLA stimulation result, in part, from the recruitment of distinct functional neuronal ensembles within the BLA.

### Memory-specific synchronization of BLA neurons by stimulation

**Fig. 4:**
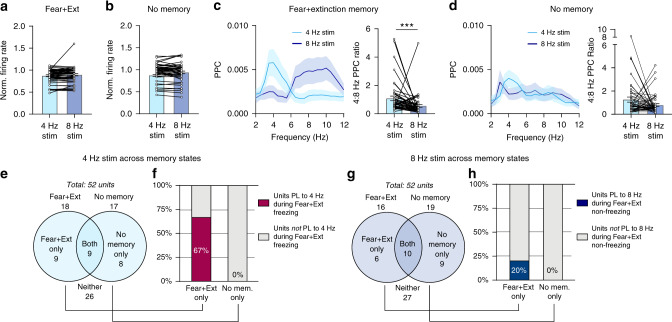
4 Hz and 8 Hz stimulation differentially synchronizes BLA neurons experience-dependently. **a** Firing rate of BLA MUA is not significantly different during 4 Hz and 8 Hz sinusoidal stimulation in the conditioned context (firing rate normalized to no light; paired two-tailed Wilcoxon test: *P* = 0.22, *n* = 50 MUA). **b** Same as in **a** but in unconditioned context (paired two-tailed Wilcoxon test: *P* = 0.33, *n* = 50 MUA). **c** Averaged PPC spectra (left) of BLA MUA during 4 Hz and 8 Hz optical stimulation in the conditioned context (Fear + Ext memory state). MUA phase-locking quantified as 4:8 Hz PPC ratio (right) is significantly shifted by 4 Hz vs. 8 Hz stimulation in the memory-context (paired two-tailed Wilcoxon test: *P* = 0.0005, *n* = 50 MUA). **d** Same as in **c** but in the unconditioned context (no memory state). MUA phase-locking is not significantly shifted by 4 Hz vs. 8 Hz stimulation in the unconditioned context (paired two-tailed Wilcoxon test: *P* = 0.17, *n* = 50 MUA). **e** Venn diagram showing the distribution of units significantly phase-locked to 4 Hz stimulus waveform in the conditioned (Fear + Ext) context (left), unconditioned neutral context (right), or both (middle overlap). **f** Proportion of units recruited to freezing-associated 4 Hz oscillations in the conditioned context is enriched among units that significantly phase-lock to 4 Hz stimulation in the conditioned context (Chi-square test: *X*^2^ = 8.24, *P* = 0.0041, *n* = 17 units). **g** Same as **e** but for 8 Hz stimulation. **h** Proportion of units recruited to non-freezing-associated 8 Hz oscillations in the conditioned context among units that significantly phase-lock to 8 Hz stimulation in the conditioned versus unconditioned context (Chi-square test: *X*^2^ = 1.94, *P* = 0.16, n = 14 units: one of the six Fear+Ext-only units in panel **g** did not meet the minimum number of spike criterium for PPC analysis during the non-freezing behavioral state). All error bars and shaded area: mean ± SEM.

We next addressed the memory-specific nature of the exogenously induced behavioral effects (Fig. 2e). For this, we first analyzed multi-unit activity (MUA) in order to obtain a broad readout of population-level spiking activity of the BLA. Similar to the effects observed in vitro (Supplementary Fig. 2), we found that the average firing rates were not different during 4 Hz and 8 Hz optical stimulation, suggesting that the bidirectional behavioral effects of 4 Hz and 8 Hz stimulation did not result from a difference in the overall firing rates of BLA neurons (Fig. 4a, b). In contrast, phase-locking of MUA was modulated differently by the two stimulations: we found that 4 Hz stimulation shifted phase-locking toward 4 Hz, while 8 Hz stimulation shifted phase-locking toward 8 Hz (Fig. 4c). Strikingly, this effect was observed only in the memory context (fear + ext), as 4 Hz and 8 Hz stimulation did not shift phase-locking towards the stimulation frequencies in an unconditioned (no memory) context (Fig. 4d). These data reveal experience-dependent changes in the ability of BLA PV- interneurons to synchronize the spiking of BLA neurons to 4 Hz and 8 Hz exogenous stimulation. This suggests that an experience-dependent neuronal resonance phenomenon, wherein resonance of spiking activity emerges after a memory-encoding experience, might support memory retrieval.

To investigate how experience-dependent resonance might support memory retrieval, we further analyzed BLA single-unit data during optogenetic stimulation. Similar to our finding that 4 Hz and 8 Hz stimulation can recruit distinct functional neuronal ensembles within the BLA (Fig. 3), we hypothesized that each stimulation might recruit distinct neuronal ensembles during different memory-states. To test this hypothesis, we analyzed BLA single-units that were recorded in both the conditioned (fear + extinction memory) context and neutral (no-memory) context. We found that 4 Hz stimulation recruited overlapping but distinct populations of units in each context (Fig. 4e). We next tested if these distinct populations differ in their phase-locking to endogenous oscillations. Interestingly, a majority of units that phase-locked to exogenous 4 Hz stimulation selectively in the conditioned context also phase-locked to freezing-associated 4 Hz oscillations in the conditioned context. In contrast, none of the units that phase-locked to exogenous 4 Hz stimulation selectively in the unconditioned neutral context phase-locked to freezing-associated 4 Hz oscillations in the conditioned context (Fig. 4f). This indicates that 4 Hz stimulation in the conditioned context recruits a functional ensemble of memory-specific freezing-associated neurons, whereas identical stimulation in the unconditioned neutral context does not recruit this ensemble. We found a similar trend for BLA single-units phase-locked to exogenous 8 Hz stimulation, although the small number of units precluded conclusive statistical evidence (Fig. 4g–h). Overall, these data reveal that the experience-dependent resonance of BLA spiking activity is associated with the recruitment of memory-specific BLA ensembles, thereby providing a mechanism by which experience-dependent resonance can support memory retrieval.

### Effects of BLA stimulation on BLA-mPFC oscillatory activity

**Fig. 5:**
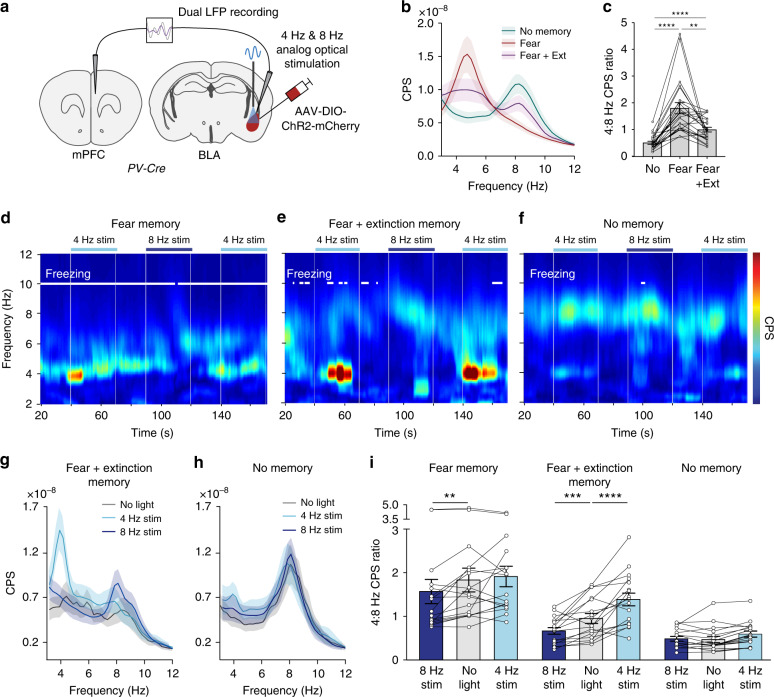
Memory-specific effects of 4 Hz and 8 Hz BLA stimulation on BLA-mPFC oscillatory activity. **a** Schematic of strategy for in vivo control of BLA PV-interneurons with simultaneous dual LFP recording. **b**, **c** Fear conditioning increases, while extinction learning reduces 4:8 Hz cross-power spectrum. Averaged cross-power spectra (**b**; *n* = 24 mice) and quantification as 4:8 Hz CPS ratio (**c**; one-way repeated measures ANOVA: *F*(1.201, 27.62) = 27.5, *P* < 0.0001, *n* = 24 mice. Tukey’s multiple comparison’s test: No memory vs. Fear memory: *P* < 0.0001; No memory vs. fear+ext memory: *P* < 0.0001; fear vs. fear+ext memory: *P* = 0.0047). **d**–**f** Representative cross-power spectrograms demonstrating bidirectional control of freezing and BLA-mPFC cross-power spectrum by 4 Hz and 8 Hz optical stimulation, exclusively during post-extinction retrieval (Fear + Extinction Memory). **g**, **h** Average BLA-mPFC cross-power spectra illustrating frequency-specific effects of 4 Hz and 8 Hz optical stimulation during the post-extinction retrieval trial (*n* = 16 mice). A stimulation-induced 4 Hz peak emerges only in the Fear+Extinction memory condition, and not in the No-memory condition. **i** Optical stimulation of BLA PV-interneurons has a bidirectional effect on BLA-mPFC cross-power spectrum in the conditioned context following, but not before, extinction learning (two-way repeated measures ANOVA: trial *F*(2,30) = 15.91, *P* < 0.0001, stimulation *F*(2,30) = 18.58, *P* < 0.0001, trial × stimulation *F*(4, 60) = 7.96, *P* < 0.0001, *n* = 16 mice; Holm-Sidak’s multiple comparisons test; fear memory: no light vs. 8 Hz stim *t*(60) = 3.29, *P* = 0.0034; no light vs 4 Hz stim *t*(60) = 0.60, *P* = 0.55; fear+ext memory: no light vs 8 Hz stim *t*(60) = 3.43, *P* = 0.0011; no light vs 4 Hz stim *t*(60) = 5.16, *P* < 0.0001; no memory: no light vs 8 Hz stim *t*(60) = 0.18, *P* = 0.86; no light vs 4 Hz stim *t*(60) = 1.43, *P* = 0.29). All error bars and shaded area: mean ± SEM.

Following our finding that the memory-specific effects of 4 Hz and 8 Hz BLA stimulation on behavior are mirrored by memory-specific synchronization of local BLA activity, we wanted to explore the possible role of other nodes in the limbic network. We decided to look at the mPFC, because oscillatory activity across BLA-mPFC circuits is correlated with various forms of fear behavior^14–19,21^, and because we previously found that 4 Hz and 8 Hz oscillatory activity across BLA-mPFC correlated with increased and decreased behavioral fear expression, respectively^20^. We simultaneously recorded LFPs from the BLA and mPFC during our behavioral paradigm (Figs. 2d and 5a). In both regions, we observed a freezing-associated oscillation centered ~4 Hz and a safety-associated oscillation centered ~8 Hz, with the relative balance between these two oscillations shifting depending on the memory state (Supplementary Fig. 4c–f). Furthermore, BLA stimulation not only shifted the BLA LFP power spectra toward the stimulation frequency, but also the mPFC LFP power spectra, though both the BLA and mPFC effects were not memory-specific (Supplementary Fig. 4d, f: no trial × stimulation interaction).

To test if the memory-specific effects of BLA stimulation on behavior could be mediated by memory-specific effects on oscillatory activity across BLA-mPFC circuits, we calculated BLA-mPFC phase coherence and cross-power spectrum (CPS). Phase coherence is a measure of the consistency of phase relationships between two oscillatory signals, and CPS is a measure of combined amplitude of oscillations across two nodes of a circuit. While high BLA-mPFC phase coherence between 3 and 12 Hz was detected throughout our behavioral paradigm, 4 Hz and 8 Hz BLA-mPFC phase coherence was not altered by learning (Supplementary Fig. 6a–d), or by 4 Hz and 8 Hz BLA stimulation (Supplementary Fig. 6i, j, l). Consistent with prior findings^19^, imaginary coherence did shift toward 4 Hz during periods of freezing (Supplementary Fig. 6e–h). Though the lack of learning- and stimulation-induced changes in phase coherence might reflect strong BLA-mPFC reciprocal coupling, technical considerations such as volume conduction limit the interpretation of our phase coherence findings.

In contrast with phase coherence, we found that BLA-mPFC CPS was distinctly modulated by fear conditioning and extinction learning, whereby BLA-mPFC CPS was shifted toward 4 Hz by fear conditioning and toward 8 Hz by extinction learning (Fig. 5b–c). We next examined whether our optogenetic BLA stimulation might also modulate this parameter. We found that 4 Hz and 8 Hz BLA stimulation shifted BLA-mPFC CPS toward the stimulation frequency, but interestingly, these stimulation effects were only observed in the conditioned context (Fig. 5d–i) and were memory-specific (Fig. 5i: significant trial × stimulation interaction). The memory-specific effects of BLA stimulation on BLA-mPFC CPS support an intriguing model whereby memory retrieval is governed by experience-dependent interregional resonance phenomena that result in the selective ability of an oscillation in one circuit node (BLA) to be amplified by another circuit node (mPFC).

### BLA-mPFC 4 Hz and 8 Hz activity predicts freezing stability

**Fig. 6:**
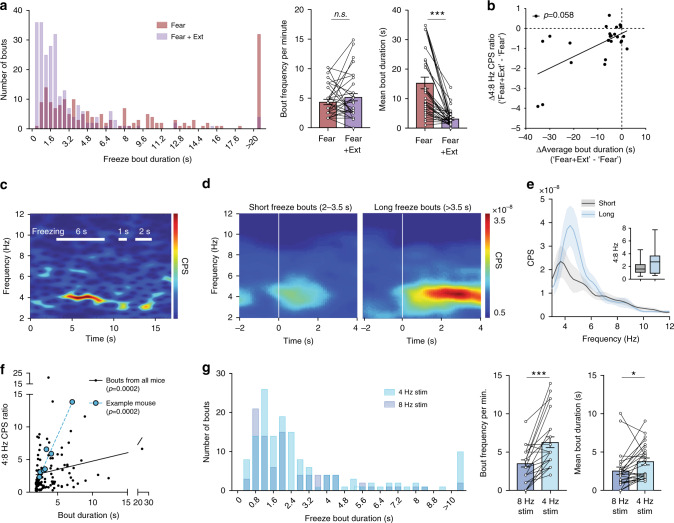
Balance between BLA-mPFC 4 Hz and 8 Hz activity predicts stability of post-extinction freezing. **a** Extinction learning reduces the duration, but not frequency, of freezing bouts. Pooled frequency histogram of all freezing bouts across all animals showed a left-shift in distribution, indicating shorter duration but not lower frequency of freezing bouts after extinction (left: *n* = 36 mice; 0.4 s bins). Comparison of pre- and post-extinction learning shows that while average bout frequency remains unchanged (middle; paired two-tailed *t*-test: *P* = 0.19, *t*(34) = 1.34, *n* = 35 mice), average bout duration is significantly reduced after extinction (right; paired two-tailed *t*-test: *P* < 0.0001, *t*(34) = 6.34, *n* = 35 mice). **b** Correlation between change in 4:8 Hz cross-power ratio with change in average bout duration (post-minus pre-extinction learning; non-parametric Spearman correlation: two-tailed *P* = 0.058; *r* = 0.39; *n* = 24 mice). **c** Example cross-power spectrogram from individual mouse showing freeze bouts of various lengths. **d**, **e** Average cross-power spectrograms (**d**) and spectra (**e**) comparing short (2–3.5 s bouts; *n* = 18 mice) vs. long (>3.5 s; *n* = 15 mice) freeze bouts during post-extinction retrieval trials. (**e** inset is quantification as 4:8 Hz cross-power ratio. Unpaired *t*-test (two-tailed): *t*(31) = 1.75; *P* = 0.09; box plot shows median (line inside box), 25% and 75% percentiles (box edges), and minimum and maximum values (error bars). **f** Correlation between the duration and the cross-power ratio of all freezing bouts (>2 s during no stimulation periods in the post-extinction retrieval trial; non-parametric Spearman correlation: two-tailed *P* = 0.0002; *r* = 0.33; 118 bouts from *n* = 23 mice). Bouts from single example mouse highlighted in blue (linear regression *F*(1,5) = 99.21, *P* = 0.0002, *R*^2^ = 0.95, *n* = 7 bouts). **g** Histogram of freezing bouts by duration during 4 Hz vs. 8 Hz stimulation in the conditioned context post-extinction learning (bouts cumulated from *n* = 25 mice). 4 Hz stimulation leads to an increase in both frequency (middle) and average duration (right) of freezing bouts compared to 8 Hz stimulation (paired two-tailed *t*-test: frequency: *P* = 0.0002, *t*(24) = 4.43; duration: *P* = 0.016, *t*(24) = 2.59; *n* = 25 mice). All error bars and shaded area: mean ± SEM.

We wanted to obtain a more detailed understanding of how shared oscillations across the BLA-mPFC network, as reflected in the BLA-mPFC CPS, might regulate the behavioral expression of competing fear and extinction memories. Closer inspection of freezing behavior episodes revealed that the predominant effect of extinction learning was a reduction in the duration of individual freezing bouts (Fig. 6a). This suggested that the primary effect of extinction learning is on the stability of the behavioral freezing state^28^. Further, there was a trend of correlation between the effects of extinction learning on 4 Hz and 8 Hz BLA-mPFC CPS and freezing bout duration (Fig. 6b). We therefore hypothesized that BLA-mPFC CPS represents a critical control parameter for the stability of the behavioral freezing state. To further investigate this possibility, we calculated BLA-mPFC CPS using the first two seconds of freezing bouts of varying duration within single post-extinction trials (Fig. 6c). We found that longer (>3.5 s) freeze bouts tended to have higher 4:8 Hz CPS within the first 2 s compared to shorter (2–3.5 s) bouts (Fig. 6d–e), and that higher initial (first 2 s) 4:8 Hz CPS ratio correlated with longer freezing bouts (Fig. 6f). Finally, we found that 4 Hz BLA PV-interneuron stimulation increased both bout duration and frequency compared to 8 Hz stimulation (Fig. 6g). Taken together, these data indicate that higher 4:8 Hz BLA-mPFC CPS, either endogenous or experimentally induced, stabilizes the behavioral expression of a fear memory as reflected by longer freezing bouts. Conversely, extinction learning appears to facilitate suppression of fear in part by reducing 4:8 Hz BLA-mPFC CPS, resulting in destabilized fear states.

### 4 Hz BLA-mPFC resonance supports memory retrieval

**Fig. 7:**
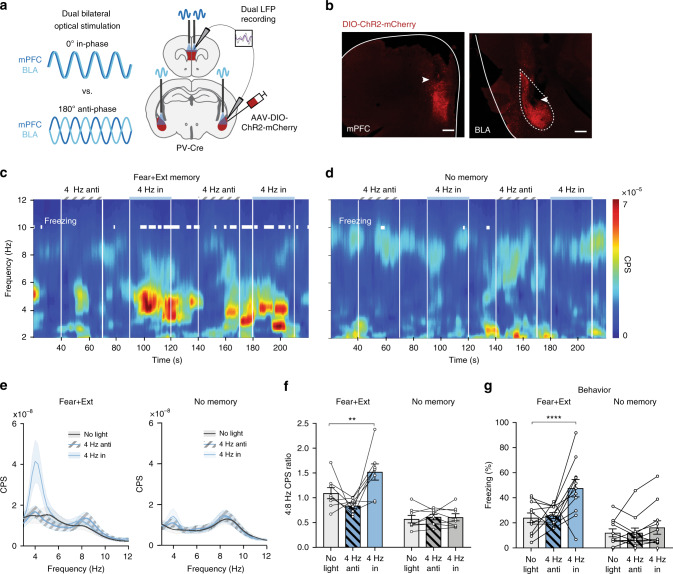
Anti-phase BLA-mPFC stimulation prevents effects of BLA-only stimulation on memory retrieval. **a** Schematic of optogenetic strategy to increase frequency-specific functional interactions between the BLA and mPFC. **b** Example images showing ChR2-mCherry expression, with optical fiber and electrode placement in mPFC and BLA. Targeting was similarly confirmed by histological analysis in all 11 mice; see Methods section for detail. Scale bar: 300 µm. **c**, **d** Representative cross-power spectrograms illustrating differential effects of 4 Hz in- and anti-phase optical stimulation in the conditioned context (**c**; fear+ext memory state) and unconditioned novel context (**d**; no-memory state). **e** Averaged cross-power spectra comparing effects of in-phase and anti-phase 4 Hz stimulation to no-stimulation baseline during fear+extinction memory and no-memory states (*n* = 8 mice). **f** Quantification of the cross-power spectra from **e**. 4 Hz in-phase stimulation increases the 4:8 Hz cross-power ratio compared to the no-stimulation baseline, whereas 4 Hz anti-phase stimulation does not. This effect is absent in a context where the mouse has no fear memory (two-way RM ANOVA: trial *F*(1,7) = 34.12, *P* = 0.0006, stimulation *F*(2,14) = 7.80, *P* = 0.0053, trial × stimulation *F*(2,14) = 7.46, *P* = 0.0062, *n* = 8 mice. Holm-Sidak’s multiple comparisons test; fear+ext memory state: no light vs. 4 Hz anti-phase: *t*(14) = 2.01, *P* = 0.064; no light vs. 4 Hz in-phase *t*(14) = 3.39, *P* = 0.0087. No memory state: no light vs. 4 Hz anti-phase: *t*(14) = 0.33, *P* = 0.94; no light vs. 4 Hz in-phase *t*(14) = 0.29, *P* = 0.94). **g** 4 Hz in-phase stimulation increases the conditioned freezing response compared to the no-stimulation baseline, whereas 4 Hz anti-phase stimulation does not. This effect is absent in a context where the mouse has no fear memory (two-way repeated measures ANOVA: trial *F*(1,10) = 15.43, *P* = 0.0028, stimulation *F*(2,20) = 10.18, *P* = 0.0009, trial × stimulation *F*(2,20) = 5.28, *P* = 0.014, *n* = 11 mice. Holm-Sidak’s multiple comparisons test; fear+ext memory state: no light vs. 4 Hz anti-phase: *t*(20) = 0.55, *P* = 0.59; no light vs. 4 Hz in-phase *t*(20) = 5.20, *P* < 0.0001. No memory state: no light vs. 4 Hz anti-phase: *t*(20) = 0.0088, *P* = 0.99; no light vs. 4 Hz in-phase *t*(20) = 0.97, *P* = 0.71). All error bars and shaded area: mean ± SEM.

Our findings thus far indicate that experience-induced changes in circuit resonance enable the transmission of frequency-specific oscillatory activity from the BLA to the mPFC, thereby supporting fear memory retrieval. We wanted to directly test such a role for experience-dependent interregional resonance across BLA-mPFC circuits. To do this, we directly manipulated BLA-mPFC oscillatory interactions through the simultaneous optogenetic manipulation of PV-interneurons in both the BLA and mPFC of mice subjected to our behavioral paradigm (Figs. 2d and 7a–b). Since the phase differences between endogenous 4 Hz oscillations in the BLA and mPFC during freezing behavior were close to 0° (Supplementary Fig. 6k), we reasoned that we could promote functional 4 Hz BLA-mPFC network states by simultaneous 4 Hz rhythmic stimulation of both BLA and mPFC PV-interneurons with a 0° (in-phase) phase relationship. On the other hand, we expected that simultaneous 4 Hz rhythmic stimulation of both BLA and mPFC PV-interneurons with a 180° (anti-phase) relationship would act as destructive interference, thereby preventing exogenously induced functional 4 Hz BLA-mPFC oscillatory interactions (Fig. 7a). These expectations align with previously reported effects of in-phase and anti-phase stimulation of human cortical regions using transcranial alternating currents^29–31^.

We found that 4 Hz in-phase stimulation of BLA and mPFC in the conditioned (fear + extinction memory) context increased 4 Hz BLA-mPFC CPS, whereas 4 Hz anti-phase stimulation in the same context had no effect (Fig. 7c, e–f). Importantly, neither in-phase nor anti-phase stimulation had an effect on BLA-mPFC CPS in the unconditioned (no memory) context, revealing the experience-dependent nature of the induced BLA-mPFC oscillatory interactions (Fig. 7d–f). Similar experience-dependent effects were observed on BLA and mPFC power spectra (Supplementary Fig. 7a–f). Interestingly, altering the relative phase of stimulation only had minor effects on BLA-mPFC phase coherence (Supplementary Fig. 7g–l). This suggests that the predominant effect of changing the phase of stimulation was on the ability of the induced oscillations to resonate across the BLA-mPFC network, as reflected by the effects on BLA-mPFC CPS. These data therefore provide further support for the occurrence of experience-dependent interregional resonance in BLA-mPFC circuits.

Behaviorally, we found that 4 Hz in-phase BLA-mPFC stimulation led to increased freezing behavior in the conditioned context (Fig. 7g), similar to the effect of 4 Hz BLA-alone stimulation (Fig. 2e). Strikingly, this behavioral effect was prevented by switching from in-phase to anti-phase stimulation, revealing that the disruption of BLA-mPFC resonance prevents the ability of oscillatory BLA stimulation to increase post-extinction fear memory retrieval. Importantly, neither stimulation condition had a behavioral effect in the unconditioned context (Fig. 7g), revealing that the behavioral effect of 4 Hz in-phase BLA-mPFC stimulation is memory-specific. Taken together, these data demonstrate that experience-dependent BLA-mPFC resonance can support BLA-mediated oscillatory control over post-extinction fear memory retrieval.

## Discussion

In this study, we examined the effects of identical oscillatory stimulations across varying memory states to interrogate experience-induced changes in the underlying circuitry. This revealed critical roles for two distinct frequency-specific network oscillations not simply in behavioral control, but in memory retrieval. Specifically, we report experience- and context-dependent control of freezing behavior via the manipulation of BLA oscillatory activity, thereby demonstrating contributions of 4 Hz and 8 Hz BLA oscillatory states to fear and extinction memory retrieval, respectively. Mechanistically, we found evidence of local and interregional resonance phenomena that were experience-dependent. At the local level, we observed an experience-dependent ability of exogenously induced oscillations to synchronize BLA spiking activity, thereby recruiting distinct functional neuronal ensembles in a memory-state specific manner. At the interregional level, 4 Hz and 8 Hz stimulation of BLA could induce 4 Hz and 8 Hz BLA-mPFC network states, but only following extinction learning and in the conditioned context. Finally, we demonstrate necessity of experience-dependent interregional resonance by preventing shared 4 Hz oscillatory activity across BLA-mPFC circuits, which prevented the ability of 4 Hz BLA stimulation to increase post-extinction fear memory retrieval. Our findings (schematized in Supplementary Fig. 8) provide several novel insights into both the functions and mechanisms of oscillatory regulation of fear and extinction memories, which we will further discuss below. In addition, our findings highlight the importance of studying oscillations not only at specific frequencies and during specific behaviors, but also across different memory states. We believe experience-dependent resonance is a concept relevant to understanding learning-dependent changes across multiple levels of the brain. Resonance potentially integrates the synaptic, cellular, and network-level changes known to contribute to learning and memory into an interpretable and manipulable parameter – a possibility on which we will further expand below.

An intriguing aspect of our findings is the experience-dependent nature of the observed effects of oscillatory BLA stimulation. The stimulation itself was identical across trials and could induce oscillatory LFP activity within the BLA regardless of experience. However, it was only after extinction, and in the conditioned context, that exogenously induced 4 Hz and 8 Hz BLA oscillations caused behavioral effects, and were reflected by increased 4 Hz and 8 Hz oscillatory power across BLA-mPFC circuits. The phenomenon whereby systems, including coupled oscillators, can amplify certain frequencies is called resonance. Our data are in agreement with an experience-induced interregional BLA-mPFC resonance that subsequently supports the retrieval of post-extinction fear memory retrieval. Although not using this terminology, a recent study demonstrated a functional role for interregional resonance in a specific limbic circuit by oscillatory stimulation of projections from the ventral hippocampus to the mPFC, which revealed that stimulation at 8 Hz, but not other frequencies, synchronized mPFC unit spiking and modulated the avoidance behavior of the animal^32^. In addition to experience-dependent interregional resonance, we also observed experience-dependent local resonance phenomena that impacted BLA unit activity. The fact that BLA resonance dynamics shifted based on the memory context indicates that these local resonance features are not epiphenomenal, but instead represent important dynamic properties relevant to amygdala function. Local inhibition-induced resonance in the theta-range has also been observed in cortical and hippocampal circuits^6^, but to our knowledge has never been described in relation to memory retrieval. Our observation of parallel local and interregional experience-dependent resonance is unlikely to be a coincidence. Our data indicate that local inhibition-induced resonance may underlie the recruitment of functionally distinct BLA ensembles during retrieval of opposing fear and extinction memories, which may be a necessary precondition for interregional resonance with the mPFC. However, it is unclear precisely how these two phenomena are related. Future work will need to more clearly delineate how experience-dependent local and interregional resonance phenomena work together to control the behavioral expression of memories.

Our data reveal that extinction learning modulates the dynamics between 4 Hz and 8 Hz oscillations across the BLA-mPFC network, such that only following extinction, 4 Hz and 8 Hz oscillations induced in the BLA can engage mPFC and drive the retrieval of post-extinction memories. Intriguingly, this is distinct from a 4-Hz oscillation induced in the mPFC, which was found to drive freezing behavior in unconditioned mice^19^. This points to a distinction between the roles of the BLA and mPFC: while oscillations generated by the mPFC appear to have executive control in driving fear responses irrespective of experience, these oscillations in the BLA can regulate fear responses only following relevant learning experiences. Our data indicate that the underlying mechanism for the BLA’s selective role in regulating learned fear is the experience-dependent ability of the BLA to resonate with mPFC oscillatory activity, suggesting that previously reported cellular and synaptic changes underlying fear and extinction learning may function to facilitate BLA-mPFC resonance. This provides an interesting avenue for future studies.

The finding that our 4 Hz and 8 Hz BLA PV stimulation only had a behavioral effect after extinction learning suggests that learning-dependent changes in the local PV network might play a role. Our unit data further support the notion that a change in the ability of BLA PV-interneurons to selectively recruit and synchronize spiking activity of specific subsets of BLA neurons to broader oscillatory activity could mediate the actual encoding of the extinction memory. Accordingly, PV interneuron networks themselves have been shown to undergo experience-induced structural and functional changes that are likely to alter local competition dynamics between distinct functional ensembles both within the BLA^33–35^, and across the BLA-mPFC network^19,20,36–38^. In this way, the BLA PV-network could act as a critical node for interpreting and filtering inputs as a result of experience-induced structural and functional changes in PV-networks^20,33,39,40^. Furthermore, this may explain why exogenous modulation of the flat, unstructured PV network that exists prior to extinction learning produced no effect on behavior. Therefore, PV-interneuron plasticity may be critical to altering or enabling meaningful communication of learned information across the BLA-mPFC network via modulation of the resonance capacity of the network.

The ability to alternate between functionally distinct oscillatory states allows a physical circuit, which is structurally static on the timescales relevant for these behavioral dynamics, to rapidly and flexibly select distinct behavioral strategies. However, a key remaining question is how the two oscillatory states lead to distinct circuit outputs. One possibility, related to points described above, is that the two oscillatory states alter the coordination of synaptic inputs and spiking output by modulating neuronal synchrony^2,41^. Our current findings indicate that 4 Hz and 8 Hz oscillations recruit distinct functional ensembles within BLA. Consistent with this, we previously found that chemogenetic inhibition of BLA PV-interneurons increased both 4 Hz BLA-mPFC oscillatory activity and the reactivation of fear-tagged ensembles of BLA and mPFC neurons^20^. The existence of subsets of neurons within the BLA and mPFC that resonate at different theta-range frequencies provides a potential mechanism for differential ensemble recruitment by 4 Hz and 8 Hz oscillatory activity^22–27^. Regarding the functional output of the circuit, it is possible that 4 Hz and 8 Hz network states differentially recruit circuit components such as the prelimbic and infralimbic mPFC, which have been reported to be pro- and anti-fear, respectively^20,42–45^. A better understanding of how oscillations differentially recruit circuit components in a rapid and flexible manner, and how memory formation biases the network to favor one oscillatory state over another, will be essential to understanding how oscillations contribute to the production of adaptive fear behavior^46,47^, and meaningful behavior in general^1^.

The context-specific nature of exogenously induced oscillatory BLA-mPFC states observed in our study suggests that a third brain region provides an additional “AND” logical gate that supports these oscillatory states in the conditioned context, but not in the unconditioned no-memory context. A strong candidate for a third brain region that confers context specificity is the hippocampus, which sends dense projections to both the BLA and PFC that regulate contextual control of fear memory retrieval^42,48,49^. Importantly, the hippocampus forms two distinct engrams for conditioned versus extinguished contextual fear^50,51^. Whether the conditioned or extinguished fear memory is retrieved might therefore be determined by a continuous functional interaction between BLA-mPFC circuits and the hippocampus, leading to the exclusive activation of either the conditioned or extinguished fear engram in the hippocampus at any point in time. Our data suggest that this process is gated by two distinct oscillatory states centered ~4 Hz and 8 Hz, which interestingly correspond to hippocampal rhythms that are observed during behavioral states of immobility and activity, respectively^52^.

Our results indicating a role for 4 Hz BLA-mPFC oscillations in stabilizing freezing bouts adds to recent reports of a respiration-synchronized 4 Hz mPFC oscillation that can similarly stabilize freezing behavior^53–56^. Based on these multiple lines of studies, we may attempt to build a general framework wherein 4 Hz respiration-synchronized oscillations can entrain limbic circuits in both an experience-independent and experience-dependent manner. Experience-independent freezing behavior, for example during the immediate response to an innately aversive stimulus, could be maintained by self-sustained 4 Hz activity through a recurrent respiration rhythm-mPFC interaction that does not require the BLA^57,58^. On the other hand, maintenance of experience-dependent freezing behavior in the absence of an innately aversive stimulus, such as during retrieval of a conditioned fear memory, would rely on the additional engagement via resonance of the BLA through 4 Hz respiration-synchronized BLA-mPFC activity. We hypothesize that a frightening experience endows a functional ensemble of reciprocally connected BLA-mPFC neurons with an intrinsic resonance to 4 Hz respiration-synchronized rhythms, possibly filtered through local PV-interneuron networks, and that such experience-induced resonance constitutes a primary determining factor in the experience-dependent control of fear behavior.

Our findings may have important translational implications. A better understanding of the neural processes leading to the return of previously extinguished fear will facilitate the design and implantation of strategies that prevent this return in patients with post-traumatic stress disorder (PTSD), or other anxiety-related disorders, who are treated with exposure therapy. Recent work in human subjects has demonstrated that fear and extinction modulate frontal and temporal lobe theta activity in a manner broadly similar to the results reported here^59^. Interestingly, single-node transcranial magnetic stimulation (TMS) of mPFC has been shown to augment the efficacy of extinction learning in humans^60^, although this study used 20 Hz pulses in PFC and did not measure its effect on network oscillations. Combined with our findings, this raises the intriguing possibility that the modulation of oscillatory activity could be used to prevent the return of extinguished fear in patients suffering from maladaptive fear. We have demonstrated that stimulation of the same population of neurons can have opposite behavioral consequences depending on the frequency of activation. One important implication of this result is that the structure of exogenous circuit activation, not just whether a circuit is activated, can be critical for recreating the salient network state and reproducing the behavioral outcome. This shows that effective manipulations can come not only from the specific activation of a sparse set of functionally defined projection neurons^61,62^, but also from a specific pattern (i.e. frequency) of activation imposed on all projection neurons within a brain region. The latter type of manipulation might be easier to adapt for use in human subjects, as the critical parameter (frequency) can be tuned non-invasively using transcranial stimulation approaches^63^. Such strategies aimed at facilitating oscillatory resonance between brain regions have recently been shown to augment working memory in older patients^31^. It will be interesting to see whether similar approaches, leveraging the experience-dependent resonance dynamics described here, can be employed to therapeutically modulate oscillatory resonance in patients suffering from pathological fear.

While our findings provide an important proof-of-principle demonstration of both the occurrence and functional significance of experience-dependent resonance in BLA-mPFC circuits, there are important remaining questions concerning the precise nature of this experience-dependent resonance. A critical feature of our experimental design was the longitudinal probing of endogenous oscillatory and behavioral responses to identical exogenous oscillatory stimulation. This design feature was essential for detecting experience-dependent resonance, which we define as the emergence of resonance at a certain frequency following a memory-encoding experience. Future studies are needed to determine the extent to which the experience-dependent resonance phenomena revealed with repeated 4 Hz and 8 Hz stimulation are associated with other changes in the resonance properties of BLA-mPFC circuits. For example, future studies can more extensively probe experience-induced changes in BLA-mPFC resonance by using a variety of additional exogenous stimulation frequencies. Additional stimulation frequencies both within and outside the theta-range are of interest. Though there is increasing support for the existence of at least two distinct functional rhythms within the 3–12-Hz theta-range in species ranging from rodents to humans^52,64–66^, additional empirical data are needed to more precisely delineate the different theta-range rhythms and their divergent functions. A variety of functionally significant rhythms are also observed outside of the theta-range, including gamma rhythms that have been directly associated with the regulation of fear behavior and memories^18,67–69^. Whether this association is also supported by experience-dependent resonance could be tested in future studies that combine our longitudinal experimental design with gamma-range oscillatory stimulation. Finally, our 4 Hz in-phase versus anti-phase experiment demonstrates that imposing constructive and destructive interference on highly localized circuit nodes can reveal the functional relevance of experience-dependent interregional resonance. Though this experimental approach is technically challenging, we believe it is worth repeating in future studies that use additional frequencies and that target other combinations of brain regions. Such experiments are expected to generate important mechanistic insights into how experience-dependent resonance supports the retrieval of memories.

## Methods

### Animals

All animal procedures were performed in accordance with the NIH Health Guide for the Care and Use of Laboratory Animals and were approved by the Tufts University Institutional Animal Care and Use Committee. *PV-Cre* mice (2–6 months old) used in this study were heterozygous for a *PV-IRES-Cre* knock-in locus (B6; 129p2-*Pvalb*^tm1(cre)Arbr^/J). Both female and male mice were used, and their data were pooled for final analysis. Mice had food and water ad libitum and were socially housed until the start of behavioral experiments, which was at an age of at least 10 weeks. Mice were kept on regular light-dark cycle, and all experiments were performed during the light phase.

### Stereotaxic surgery

Mice were anesthetized with isoflurane, held in a stereotaxic apparatus (Kopf) and injected with virus. After injection, the needle was left in place for 10 min before being slowly retracted. The incision was sutured, and mice were weighed and monitored to ensure recovery. For BLA optogenetic stimulation experiments, 250 nl of AAV-Syn-DIO-ChR2-mCherry or AAV-Ef1a-DIO-hChR2(H134R)-mCherry (UNC Vector Core, Karl Deisseroth) was injected into BLA (AP − 1.35, ML ± 3.45, DV − 5.15 mm). Most mice in this experiment were injected and targeted unilaterally, except for five mice that were targeted bilaterally. Data from unilateral and bilateral mice were pooled for final analysis, as similar effects were observed in both groups.

Mice were implanted with fiber optic cannulae at 2–3 weeks following virus injection (Thorlabs, CFM12L05) in the BLA (AP − 1.35, ML ± 3.45, DV −5 mm). During this surgery, mice that required LFP recordings were implanted with electrodes (PFA-coated tungsten wire; AM systems) in the BLA (AP − 1.35, ML ± 3.45, DV −5 mm) and mPFC (AP + 1.75, ML ± 0.3, DV −2 mm). The two electrodes were attached to prefabricated headmounts (Pinnacle; #8201). The headmounts were affixed to the skull with stainless steel screws that also act as EEG reference and ground electrodes placed in the cerebellum. Headmounts were connected to a 100× preamplifier (Pinnacle; 8202-SE). For sham surgeries, mice were implanted with optical cannulae in an identical fashion, but without expression of ChR2 (either because of no viral injection, or because of no *PV-Cre* transgene).

For experiments involving stimulation of both BLA and PFC, the same procedures were followed as above but with injections and fiber implants made bilaterally in to the BLA and PFC (AP + 1.75, ML ± 0.3, DV for virus −2.3 mm, DV for fiber −2 mm), and electrodes implanted unilaterally in the right BLA/PFC (same coordinates as optic fiber).

### Behavior

Behavior started 1 week after the implantation of fibers and electrodes, and at least 3 weeks after virus injection. None of the mice had prior procedures or testing performed, and mice were randomly assigned to experimental groups. Mice were subjected to contextual fear conditioning consisting of three training trials (FC1, FC2, and FC3) with 3 h between each trial. The total duration of each training trial was 500 s. A training trial started with placing the mouse in a square chamber with grid floor (context A; Coulbourn Instruments; H10-11RTC). At 240, 300, 360, and 420 s, a foot shock was delivered (2 s and 0.70 mA). On days 2 and 3 (or 4 and 5 for the delayed extinction group), mice were subjected to a maximum of four extinction trials per day. Post-FC retrieval was performed at the start of the first extinction trial. Each extinction trial lasted 1200 s, with an inter-trial interval of 2 h. For each extinction trial, mice were placed in the same box used for fear conditioning without receiving foot shocks. Extinction training ended either after 8 trials or when the animal exhibited freezing levels <20% at the start of an extinction trial, whichever came first. This was done to avoid a potential floor effect after extinction learning so that we could adequately assess possible freezing-reducing effects of optical stimulation. Following extinction (on day 4 for standard extinction group and day 6 for delayed extinction group), mice were tested over 240 s during a single retrieval test in context A. If freezing during this trial exceeded 50% of Post-FC freezing levels, indicating inadequate extinction learning, data from that retrieval trial were discarded and the retrieval trial was performed again on the following day with identical parameters. Following the context A retrieval trial and on the same day, mice underwent a 240-s retrieval trial in context B, which consisted of a square plastic box with bedding sprayed with 10% acetic acid and striped walls.

### In vivo optical stimulation

A 4-Hz and 8-Hz analog sinusoidal stimulation protocol was designed in LabChart software (except see ‘In vivo single- and multi-unit recordings and analysis’ section) and fed through to a laser (Laserglow, LRS-0473 DPSS Laser). For the BLA + mPFC dual stimulation experiment (Fig. 7), two separate lasers controlled the stimulation of BLA and mPFC, such that the one laser stimulated either in-phase (in-phase condition) or with an 180 degrees phase-shift relative to the other laser (anti-phase condition). Laser output at fiber tip at the peak of sine wave was ~10 mW. Optical stimulation was performed during the first extinction trial, the retrieval trial in context A and the context B trial. In addition, optical stimulation was performed during the first fear conditioning trial in order to habituate the mice to the sudden appearance of light. For all trials, optical stimulation was performed during four 30 s intervals (40–70 s, 90–120 s, 140–170 s, and 180–210 s). These four stimulation periods alternated between 4 Hz and 8 Hz, or in-phase and anti-phase. For each animal, the order of stimulation was consistent across trials, but the order was varied (4-8-4-8 vs. 8-4-8-4; in-anti-in-anti vs. anti-in-anti-in) across animals to ensure absence of order-dependent effects.

### Quantification of freezing behavior

Freezing behavior was recorded using a digital camera connected to a computer with Actimetrics FreezeFrame software. Freezing behavior was quantified by human observer, because software-enabled quantification was not possible due to interference of light stimulation with automated video analysis. Quantification of freezing behavior was performed by individuals blinded to the experimental design and order of stimulation. No-light freezing scores were calculated during the epochs in which the light was off (from 0–40 s and 210–240 s) and optical stimulation freezing scores were calculated during the 4- and 8-Hz stimulation (each 2x 30 s per behavioral trial). All freeze bouts longer than 0.5 s were included in quantification (except in the analysis of short vs. long freeze bouts and correlation of bout duration with CPS), when only bouts longer than 1.9 s were included).

### In vitro electrophysiology

After decapitation under isoflurane anesthesia, the brain was quickly removed from the skull and placed in ice-cold artificial cerebrospinal fluid (aCSF) saturated with 95% O2 and 5% CO2 and containing 2 mM Mg2+ and 1 mM Ca2 + . Coronal slices (350 µm) containing the amygdala, and approximately spanning Bregma −1 to −2.5 mm, were obtained with a Leica VT1200s vibratome, incubated 35 min at 33 °C in 1.5 mM Mg2+ and 2 mM Ca2 + -containing aCSF and then allowed to recover for 45 min at room temperature. Slices were then transferred into a recording chamber, where they were perfused with aCSF (~2 mL/min) saturated with 95% O2/5% CO2 at 34 °C (temperature controller TC344B Warner instrument Co.). The aCSF composition was (in mM): 120 NaCl, 3.2 KCl, 1 NaH2PO4, 26 NaHCO3, and 10 glucose (pH 7.3, 290-300 mOsm.L-1). mCherry-positive PV interneurons were identified using a Nikon fluorescence microscope (Eclipse FN-1). They were patched using an infrared camera and DIC system (DAGE-MTI) under a ×16 objective. Patch-clamp recording pipettes (3–6 MΩ) were filled with a potassium gluconate solution containing (in mM): 130 K+-gluconate, 2 KCl, 10 HEPES, 3 MgCl2, 2 K-ATP, 0.2 Na-GTP, 5 Phosphocratine di, tris (pH 7.3, 290 mOsm.l-1). Cells with an access resistance >25 MΩ were immediately discarded. Whole-cell patch-clamp recordings were obtained in current-clamp mode, and cells were held at their resting membrane potential throughout (Iinj = 0 pA). The optical stimulation was delivered from a 430/490 nm DPSS laser system (LaserGlow) through an optic fiber placed in the bath immediately above the slice and connected by a bridge cable (400 µm, 0.39 NA, Thorlabs). The light power was measured at the tip of the optic fiber prior to experiments. The laser was controlled by an Agilent 33,210 A analog waveform generator, also connected to the Digidata 1322A Analogue In for offline analysis, and delivering analogue sinusoidal stimulations of desired frequency (Vpp 1 V; Offset 0 V). The laser output power and the waveform stimulation frequency were switched manually between stimulations. Each cell received both 4 Hz and 8 Hz stimulations (separated by at least 30 s) at incremental light powers of 1, 10, 75, 220, 375, and 400 µW, at least once over the course of the 15–30-min recording. Data were recorded with a Multiclamp 700B amplifier (Axon Instruments, Inc.) through a Digidata 1322A, sampled at 20 kHz, filtered at 10 kHz, and analyzed using pClamp10 software (Axon Instruments, Inc.).

### In vivo LFP recordings and analysis

Electrophysiological activity was acquired using the Powerlab Labchart system (ADI instruments) at 4 kHz. Data were analyzed using custom MATLAB scripts utilizing functions available in the Fieldtrip and EEGLAB toolboxes. Raw LFPs were down-sampled to 1000 Hz and bandpass filtered between 0.7 and 300 Hz. Spectral analysis was conducted by applying the Hanning taper method with 1.5 s windows and 95% overlap. Cross-power spectrum (*CPS*) was calculated as the averaged absolute value of the cross-spectrum $( {S_{{\mathrm{xy}}}} )$ between BLA and PFC: $CPS = \frac{{{\mathrm{\Sigma }}\left| {S_{{\mathrm{xy}}}} \right|}}{N}$. Coherence (*coh*) and imaginary coherence (*iCoh*) between BLA and PFC LFPs was calculated as:

$$coh_{{\mathrm{xy}}} = \frac{{\left| {{\sum} {S_{{\mathrm{xy}}}} } \right|}}{{\sqrt {\mathop {\sum}\nolimits S_{{\mathrm{xx}}} \times \mathop {\sum }\nolimits S_{{\mathrm{yy}}}} }}\;\;\;\;\;\;iCoh_{{\mathrm{xy}}} = \frac{{\left| {imag\left( {\mathop {\sum }\nolimits S_{{\mathrm{xy}}}} \right)} \right|}}{{\sqrt {\mathop {\sum }\nolimits S_{{\mathrm{xx}}} \times \mathop {\sum }\nolimits S_{{\mathrm{yy}}}} }}$$

All power, cross-power, and coherence spectra were quantified as a ratio of 3–6:6–12 Hz area-under-curve (referred to in text as “4:8 Hz Power/Cross-power/Coherence ratio”) using GraphPad Prism.

For analysis of stimulation periods, CPS and coherence spectra for every stim condition, for each mouse, was calculated from the two stimulation (or no light) periods in each trial. For analysis of CPS or coherence measures across memory states, LFPs from no light periods during each trial were used. For analysis of phase differences between PFC and BLA, LFPs were first bandpass filtered to 3–5 Hz for the 4-Hz oscillation and 7–9-Hz for the 8 Hz oscillation. From the Hilbert transform of these signals, the instantaneous phases at each time point were calculated. Phase difference was calculated as ${\mathrm{phase}}_{{\mathrm{PFC}}} - {\mathrm{phase}}_{{\mathrm{BLA}}}$.

For analysis of short vs. long freeze bouts, every mouse with at least two short (2–3.5 s) or two long (>3.5 s) freeze bouts was included. The CPS was then calculated using the first two seconds of these bouts and averaged for each mouse.

### In vivo single- and multi-unit recordings and analysis

Custom-made opto-tetrodes twisted from four 12.7-µm hard PAC coated Nickel-Chrome wires (Sandvik, FL). For each mouse, a 3D-printed tetrode drive (tough resin) was used to host up to eight tetrodes^70–72^. Each tetrode wire was attached to the electrode interface board (EIB 32 channels with Omnetics connector, Open-Ephys) by an EIB pin (Large pin, Neuralynx). Tetrodes were then fixed to a 200-µm optic fiber (ceramic cannulae, Thorlabs or RWD) with super glue (Loctite). Tetrodes were cut at a 45 degree angle at 300–500 µm below the fiber tip such that each tetrode had varying lengths. Tetrodes were gold plated (Gold Non-Cyanide solution, Sifcoasc) to reduce the electrode impedance to 250 KΩ at 1 kHz (nanoZ, Plexon). Tetrodes were implanted into the BLA as described above (see “Stereotaxic surgery”). Mice were allowed to recover for 1 week and were habituated to handling during that time. Electrolytic lesion was performed before brain dissection to mark the electrode tip location by injecting a 30-s constant 30 µA current into each selected EIB pin (A365 stimulus isolator, WPI).

The behavioral protocol is as described above with the exception that a total of 4 min of sinusoidal optical stimulation was performed (eight 30-s stimulation periods alternating between 4 Hz and 8 Hz) per behavioral trial in order to increase sampling of potential infrequently-spiking units. In addition, 30 min of opto-tagging stimulation (15 ms square wave pulses, 0.5 Hz) was performed in a neutral context following the completion of the retrieval trials on the final day.

Multichannel neural signals were recorded, amplified, and digitized with a RHD2132 amplifier board (32 unipolar channels with 3-axis accelerometer, Intan Technologies) which was controlled by the Open-Ephys Acquisition Board^73^. Raw data were sampled at 30 kHz and bandpass filtered between 0.3 and 8850 Hz. For online spike detection, raw data were further bandpass filtered between 700 and 7000 Hz and the amplitude threshold of spike waveforms for each channel was manually selected to ensure most of spike events are detectable. Custom-written Matlab scripts were used to control the analog light pattern of a 473-nm DPSS laser (Laserglow Technologies) via a USB data acquisition device (USB-1208FS-Plus, Measurement Computing Corporation). The analog light waveform, behavioral paradigm related event markers were all recorded by Open-Ephys.

Spikes were detected online and sorted offline. For analysis of single-units, semi-automated spike waveform clustering was performed using KlustaKwik (K. Harris) and MClust-4.0 (A.D. Redish) based on waveform features energy, valley, and wavePC1. Clusters were then manually refined to isolate out single-units based on waveforms, auto-correlogram, inter-spike-interval (ISI) histogram, and cluster quality metrics (isolation distance (ID) and L-ratio)^74^. Clusters with L-ratio <0.5, ID >12, and <0.5% of ISI shorter than 2 ms were classified as single-units (for all single-unit clusters, the median L-ratio was 0.07, the median ID was 23, and the median percentage of ISI < 2 ms was 0.1%). For analysis of multi-units, all spikes detected by a single tetrode were considered a single MUA.

For the analysis of phase-locking of BLA units to endogenous 4 Hz and 8 Hz oscillations, we analyzed a continuous extinction trial during which both 4 Hz and 8 Hz oscillations are observed as the mice alternate between freezing and non-freezing behavior. This allowed us to follow the activity of the same units during both behaviors. To ensure sufficient sampling, a 350–600-s period was analyzed for each condition/mouse. The strength of spike-LFP phase-locking was assessed by calculating the pairwise-phase consistency (PPC) measure using the Fieldtrip toolbox in Matlab^75,76^. Briefly, for every given frequency (*f*), the phase of each spike relative to the LFP was determined by cutting out a 5/*f* LFP epoch length which was then Hanning window tapered and Fourier transformed. To avoid spike contamination of PPC, LFP of the channels from which the unit was detected were excluded. For each unit, the final PPC is an average across LFP channels. To minimize variability in the estimation of PPC (Vinck et al.^75^), units that did not have at least 90 spikes during the analyzed time period were excluded. To test for statistical significance of PPC, we performed permutation testing for each unit in every frequency in the 3–12-Hz range. The LFP signal was cut into 1 s segments, which were then randomly shuffled to generate a surrogate reference LFP. For each randomization iteration (*N* = 100), PPC was calculated for each frequency between 3 and 12 Hz, which constituted a reference distribution for statistical test. We then determined if the tested value lies above the 95th percentile of the reference distribution, in which case, we considered the unit to be significantly phase-locked to a given frequency. Units that significantly phase-locked to either 3–5 Hz or 7–12 Hz were classified as phase-locking to 4 Hz or 8 Hz, respectively. Some units showed significant phase-locking in both or neither frequency ranges, and were classified as such. By comparing this classification for each unit during freezing and non-freezing periods, we identified units that phase-locked exclusively to 4 Hz or 8 Hz, as well as those that switch.

For the analysis of BLA single-unit spiking activity during 4 Hz and 8 Hz stimulation, a total of 120 s per stimulation frequency was analyzed (4 × 30 s stimulation epochs per stimulation frequency, as described above). Phase-locking to stimulus waveform for each unit with at least 30 spikes was analyzed by calculating PPC to the stimulus waveform, and testing significance to allow classification of units as significantly phase-locking to 4 Hz or 8 Hz stimulation or not. The phase-locking preference of these same units during endogenous activity was also analyzed by calculating PPC to LFP, only for those units with at least 70 spikes either during freezing and non-freezing behavior (during periods of no stimulation). This phase-locking preference was quantified as the ratio of the area under the curve of the PPC spectra between the 4-Hz band (3–6 Hz) and 8-Hz band (6–12 Hz; referred to in figures as ‘4:8 Hz PPC ratio’).

Similarly, for the analysis of phase-locking of BLA MUA during 4 Hz and 8 Hz stimulation, the same 120 s of stimulation period was analyzed for both the ‘fear+extinction memory’ and ‘no memory’ retrieval trials. PPC to ongoing LFP was calculated and quantified as ‘4:8 Hz PPC ratio’ for each MUA as described above.

### Histological analysis

Mice were deeply anesthetized and intracardially perfused with 0.1 M phosphate buffer (PB) followed by 4% paraformaldehyde (PFA 4%) dissolved in 0.1 M PB. Brains were extracted and post-fixed in PFA 4% for 24 h. Brains were transferred to 30% sucrose for 48–72 h before slicing 30-μm coronal sections of the entire brain using a cryostat. Sections were stored in phosphate-buffered saline (PBS) with 0.025% sodium azide at 4 °C until use. For immunofluorescence staining, sections were blocked for 1 h at room temperature in PBS-T (PBS with 0.25% Triton X-100) with 8% normal goat serum. Sections were incubated in mouse anti-PV (Millipore MAB1572; monoclonal; 1:2000) at 4 °C for 48–72 h. The secondary antibody (ThermoFisher, Alexa Fluor goat anti-mouse 488, 1:1500) was diluted in the blocking solution and were then applied to the sections for 1 h at room temperature, followed by three rinses for 15 min in PBS-T. Sections were mounted on slides and coverslipped. A wide-field epifluorescence microscope (Keyence BZ-X700) was used to acquire images for electrode and injection site validation. Images were obtained at 10–20× and stitched together using Keyence software. A confocal laser-scanning microscope (Leica SPE) was used to acquire images for mCherry/PV overlap analysis.

For the analysis of ChR2-mCherry expression in the piriform cortex, mean mCherry fluorescence intensity in the BLA and piriform cortex was quantified using ImageJ. Each value was then normalized to the background fluorescence level in the same section (mean fluorescence intensity in the adjacent/somatosensory cortex). The ChR2-mCherry expression level in the piriform cortex was calculated as the percentage of normalized mean fluorescence intensity in the piriform cortex relative to that in the BLA (separately calculated for approximately eight sections per mouse, followed by calculating the average percentage for each mouse).

### Statistical analysis

Statistical tests were performed using Prism (GraphPad) and are indicated in the figure legends. All statistical tests were two-tailed. All error bars and shaded area in graphs are standard error of means (SEM).

### Exclusion of mice from analysis

Mice were excluded from all analysis (behavioral and electrophysiological) if they had insufficient ChR2-mCherry expression within BLA/mPFC, or if expression was not targeted to the BLA/mPFC. Mice were excluded from electrophysiological analysis if electrode placement was outside of the targeted region (BLA/mPFC), or if recordings were contaminated with noise such as motion artifacts, or if there was no prominent 4 Hz oscillation detected following fear conditioning; of these mice, a subset were included only for behavioral analysis if targeting of virus and optic fibers were correct. Exclusion of mice was determined blind to experimental conditions.

### Reporting summary

Further information on research design is available in the Nature Research Reporting Summary linked to this article.

## Supplementary information

Supplementary Information

Reporting Summary

## Data Availability

Source data are provided with this paper.

## Source data

Source Data

## Code availability

Data were analyzed using functions available in the Fieldtrip and EEGLAB toolboxes with minor customizations in MATLAB. Customized scripts used in this study are available from the corresponding author upon reasonable request.
