# Supplementary material for: Experience-dependent resonance in amygdalo-cortical circuits supports fear memory retrieval following extinction
Source: Nat Commun. 2020 Aug 31;11:4358. doi: 10.1038/s41467-020-18199-w (PMC7459312; doi:10.1038/s41467-020-18199-w)
Supplement: Supplementary file 1 — Supplementary Information [file 41467_2020_18199_MOESM1_ESM.pdf]

Supplementary Information

Experience-dependent resonance in amygdalo-cortical circuits supports fear memory retrieval following extinction

Minagi Ozawa*, Patrick Davis*, Jianguang Ni, Jamie Maguire, Thomas Papouin, and Leon Reijmers

* These authors contributed equally

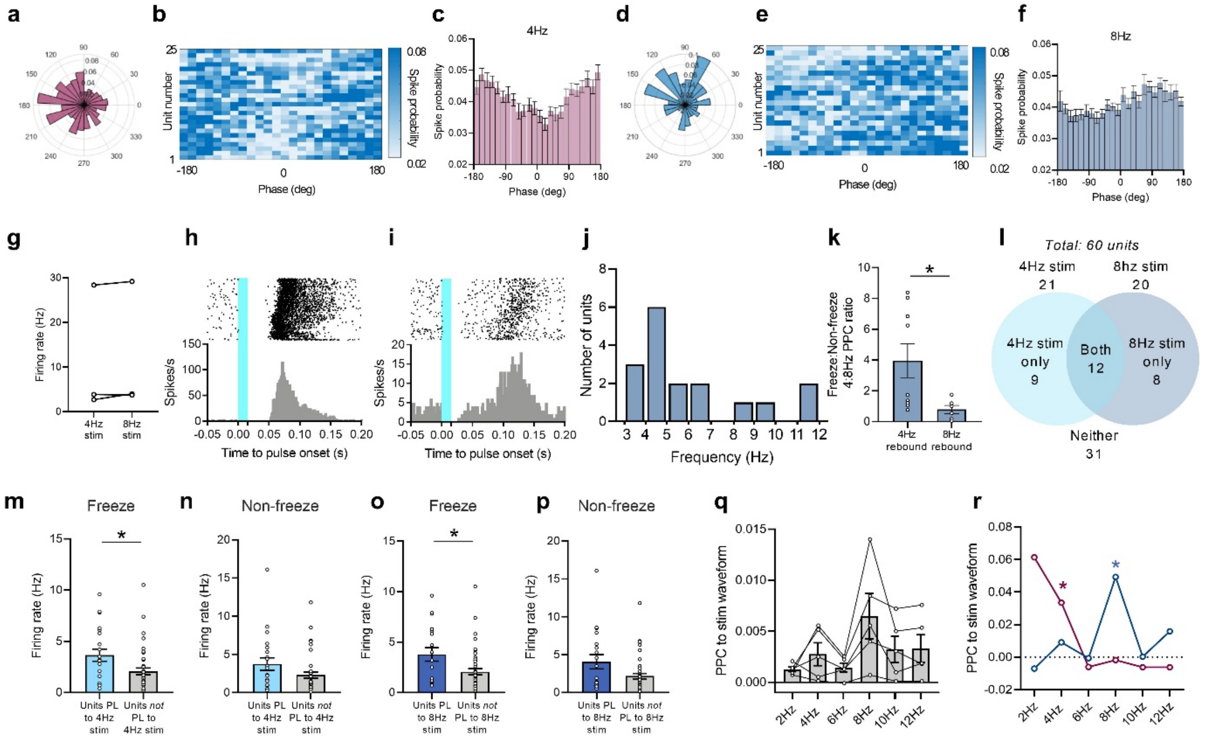

Supplementary Figure 1. Properties of BLA single units.

(a) Distribution of spike probability relative to the phase of a 4Hz oscillation for an example phase-locked BLA unit during freezing. **(b)** Distribution of spike probabilities relative to the phase of 4Hz oscillation for all units significantly phase-locked to 4Hz during freezing (n=25 units). **(c)** Average spike probability distribution relative to the phase of 4Hz oscillation for all units significantly phase-locked to 4Hz during freezing (n=25 units). **(d)** Distribution of spike probability relative to the phase of an 8Hz oscillation for an example phase-locked BLA unit during non-freezing. **(e-f)** Same as in (b-c) but relative to 8Hz oscillation during non-freezing (n=22 units). **(g)** Firing rate of light-activated units during 4Hz and 8Hz stimulation. **(h-i)** Raster plot (top) and spike histogram (bottom) of two example light-suppressed BLA single-units showing rebound activity of different kinetics after onset of a 15ms light pulses. Unit in (h) shows peak rebounding at around 72 ms, whereas unit in (i) at around 115 ms. (j) Distribution of units by rebounding kinetics: peak rebound time was assigned to an oscillation frequency based on corresponding half time of period (n=17 units). **(k)** Rebounding units corresponding to 4Hz vs. 8Hz have a differential preference for phase-locking to 4Hz or 8Hz during freezing and non-freezing (Mann-Whitney test, two-tailed: U=8, P=0.026, n=9 and 6 units). **(l)** Venn diagram showing the distribution of units significantly phase-locked to 4Hz stimulus waveform (left, light blue), 8Hz stimulus waveform (right, dark blue), or both (middle overlap) in the conditioned (Fear+Ext) context. **(m)** Firing rate during freezing of units that significantly phase-locked to 4Hz stimulation vs. those that did not (Mann-Whitney test, two-tailed: U=293, P=0.022, n=22 and 41 units). **(n)** Same as in (m) but during non-freeze periods (Mann-Whitney test, two-tailed: U=308.5, P=0.12, n=21 and 39 units). **(o)** Firing rate during freezing of units that significantly phase-locked to 8Hz stimulation vs. those that did not (Mann-Whitney test, two-tailed: U=273, P=0.022, n=19

and 45 units). **(p)** Same as in (o) but during non-freeze (Mann-Whitney test, two-tailed: U=284, P=0.074, n=19 and 42 units). **(q)** Phase-locking strength of BLA MUA to various sinusoidal stimulation frequencies (n=5 MUA from 1 mouse). **(r)** Two example BLA single units that show preference for significantly phase-lock to 4Hz or 8Hz stimulation waveform, but not other frequencies (significance based on permutation testing p>0.05; unit at 4Hz: P=0.029; 8Hz unit P=0.018). All error bars: mean ± SEM.

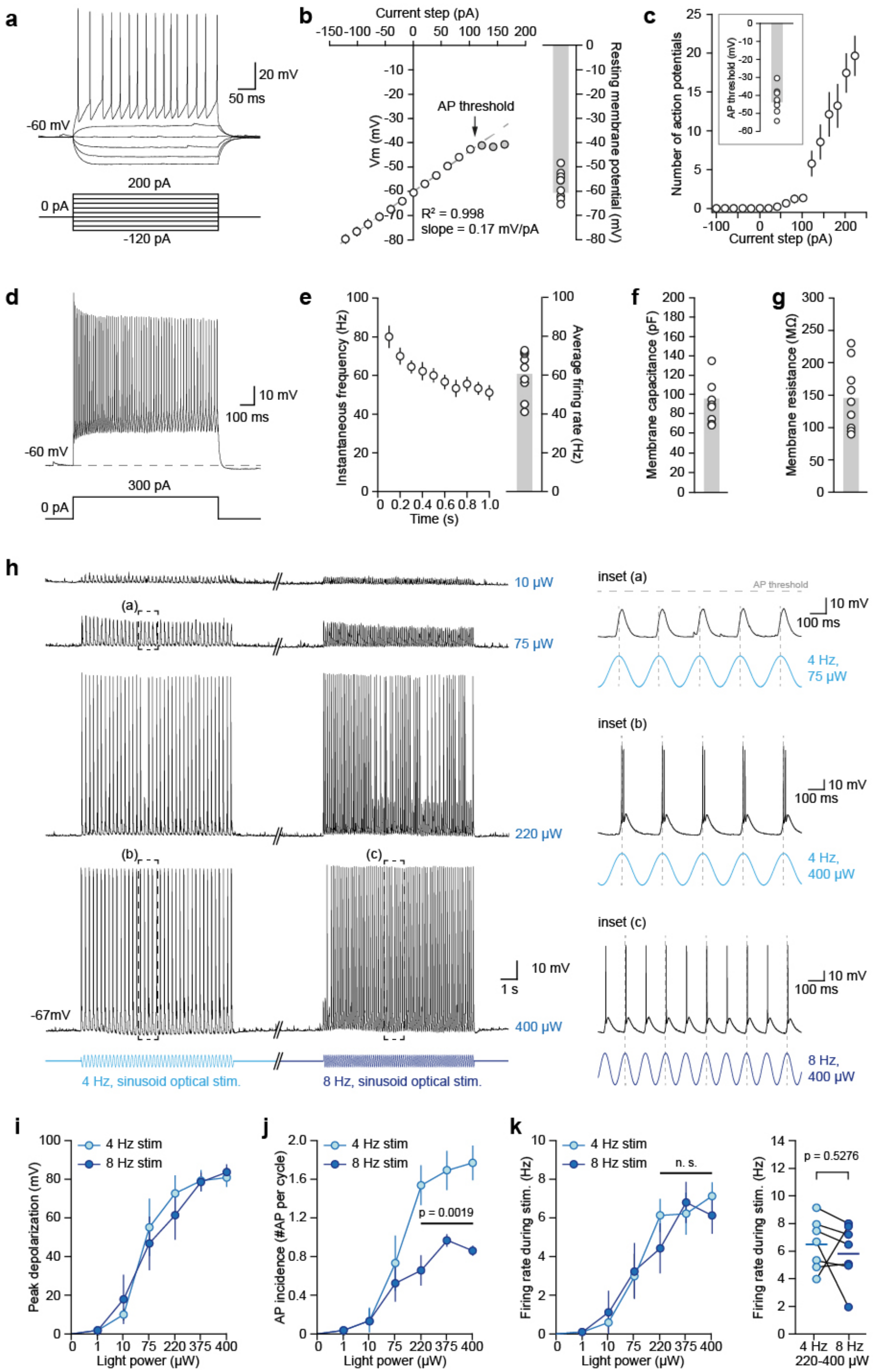

Supplementary Figure 2. Optical stimulation of BLA PV-interneurons expressing ChR2-mCherry.

(a) Voltage response of a BLA PV interneuron to 400 ms hyperpolarizing and depolarizing current steps (-120pA to 200pA; 20pA increments). All neurons were held at their resting membrane potential. **(b)** Current-voltage (I-V) curve of BLA PV interneurons for current steps of -120 to

+160 pA (left), and resting membrane potential (right, -60.8 ± 1.8 mV, n = 9 cells). **(c)** Current-firing curve showing the number of action potentials (APs) evoked in response to 400 ms current steps (-120pA to 200pA; 20pA increments). Inset shows the membrane potential threshold for AP firing (-44.1 ± 1.5 mV, n = 9 cells). **(d)** AP firing and adaptation response to a 1 s, 300 pA current step. All neurons were held at their resting membrane potential. **(e)** Time course of the average instantaneous AP firing frequency during a 1 s, 300 pA depolarizing step as shown in d (left) and corresponding mean firing rate over the entire current step (right, 60.7 ± 3.9 Hz, n = 9 cells). **(f-g)** Scattered plots of the membrane capacitance (f, 94.1 ± 6.3 pF, n = 9 cells) and input resistance (g, 146.8 ± 17.2 MΩ, n = 9 cells) of BLA PV-interneurons expressing ChR2-mCherry. **(h)** Example of the voltage response of a BLA PV-interneuron expressing ChR2-mCherry, held at resting membrane potential, to 10 s sinusoidal optical stimulations at 4 Hz and 8 Hz for increasing light powers (10, 75, 220 and 400 µW shown here). 4 Hz and 8 Hz optical stimulations were delivered at least 30 s apart. Insets show enlarged epochs of the voltage response during optical stimulation: inset (a) shows the response to an infra-threshold 75 µW, 4Hz stimulation; insets (b) and (c) show the firing response for 4 Hz and 8 Hz optical stimulations respectively (power: 400 µW). Note the reliability and phase-locking of the response, and the identical number of APs fired over the entire epoch (also see panels j and k). **(i)** Summary graph showing the mean peak membrane depolarization in response to 4 Hz (light blue) and 8 Hz (deep blue) optical stimulations of increasing power (1 to 400 µW). The mean peak membrane depolarization was obtained by averaging the maximum voltage response at each cycle of the sinusoid over the 10 s optical stimulation (paired Student's t-test: two-tailed P=0.0019, n= 7 cells). **(j)** Summary graph showing the number of APs fired per cycle at 4 Hz (light blue) and 8 Hz (deep blue), for increasing intensities of optical stimulation (also see insets a-c of panel h). For supra-threshold stimulations (220 to 400 µW), the number of AP fired during each cycle at 4 Hz (1.67 ± 0.17) is 2.0 ± 0.2 times that at 8 Hz (0.84 ± 0.06). The number of APs fired per cycle was averaged across 220-400 µW stimulations for each cell (n= 7 cells). **(k)** Left, summary graph showing the average firing rate over the entire 10 second optical stimulation at 4 Hz (light blue) and 8 Hz (deep blue) for increasing power of optical stimulation. Right, paired data showing the absence of difference between the overall firing rate at 4 Hz (6.5 ± 0.7 Hz) and that at 8 Hz (5.8 ± 0.9 Hz). The firing rate was averaged across 220-400 µW stimulations for each cell. Note that while cells fire on average twice as many APs per cycle during the 4 Hz stimulation compared to the 8 Hz stimulation (see insets b-c of panel h, and see panel j), the overall firing rate is normalized over the 10 s stimulation epoch since the 8 Hz stimulation contains twice as many cycles as the 4 Hz stimulation (paired Student's t-test: two-tailed P=0.53, n=7 cells). All error bars: mean ± SEM.

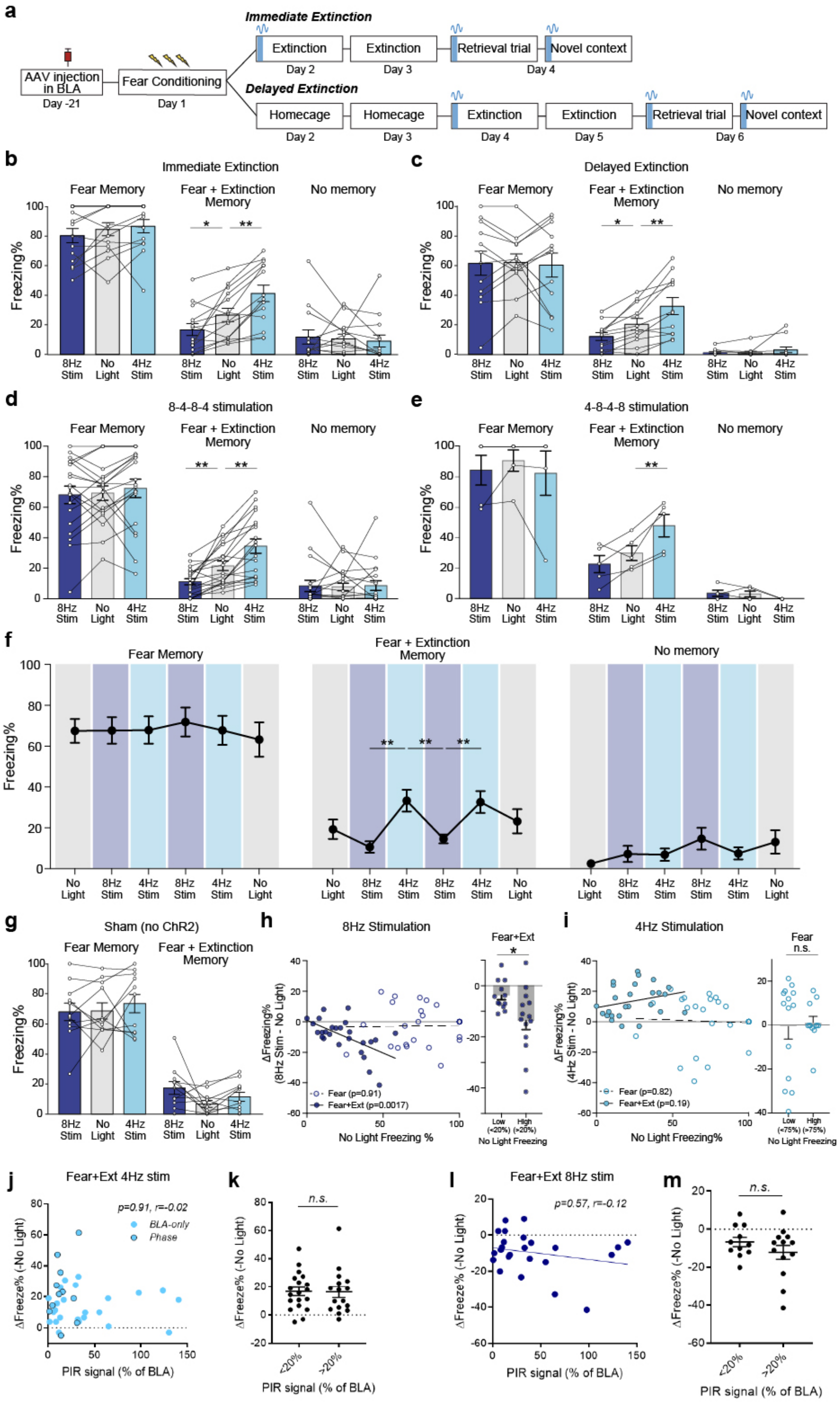

Supplementary Figure 3. Specificity of the behavioral effects of BLA PV-interneuron stimulation.

(a) Schematic of standard and delayed extinction behavioral paradigms. For standard extinction, mice were fear conditioned on day 1, subjected to extinction learning on days 2-3, and retrieval

on day 4. For delayed extinction, mice were fear conditioned on day 1, left in their homecage on days 2-3, subjected to extinction learning on days 4-5, and retrieval on day 6. **(b-c)** The behavioral effects of BLA PV-interneuron stimulation specifically rely on the memory-state, and not the passage of time. Neither the standard- nor delayed extinction group demonstrated an effect of stimulation prior to extinction learning (fear memory state) or in a novel context (no memory state), whereas both groups showed bidirectional effects of stimulation following extinction learning (fear + extinction memory state) (two-way repeated measures ANOVA: Standard Extinction: trial F(2,26)=93.62, P<0.0001, stimulation F(2,26)=16.4, P<0.0001, trial x stimulation F(4, 52)=5.02, P=0.0017, n=14 mice; Holm-Sidak's multiple comparisons test; Fear Memory: no light vs. 8Hz stim t(52)=0.97, P=0.56; no light vs 4Hz stim t(52)=0.47, P=0.64; Fear+Ext Memory: no light vs 8Hz stim t(52)= 2.25, P=0.029; no light vs 4Hz stim t(52)=3.30, P=0.0035; No Memory: no light vs 8Hz stim t(52)=0.26, P=0.93; no light vs 4Hz stim t(52)=0.35, P=0.93; Delayed Extinction: trial F(2,22)=49.57, P<0.0001, stimulation F(2,22)=4.67, P=0.020, trial x stimulation F(4, 44)=6.37, P=0.0004, n=12 mice; Holm-Sidak's multiple comparisons test; Fear Memory: no light vs. 8Hz stim t(44)=0.23, P=0.82; no light vs 4Hz stim t(44)=0.57, P=0.82; Fear+Ext Memory: no light vs 8Hz stim t(44)= 2.51, P=0.016; no light vs 4Hz stim t(44)=3.67, P=0.0013; No Memory: no light vs 8Hz stim t(44)=0.02, P=0.98; no light vs 4Hz stim t(44)=0.58, P=0.81). **(d-e)** The behavioral effect of BLA PV-interneuron stimulation was similar for both stimulation orders (two-way repeated measures ANOVA: 8-4-8-4 stimulation order: trial F(2, 36)=97.88, P<0.0001, stimulation F(2, 36)=17.1, P<0.0001, trial x stimulation F(4, 72)=6.04, P=0.0003, n=19 mice; Holm-Sidak's multiple comparisons test; Fear Memory: no light vs. 8Hz stim t(72)=0.34, P=0.74; no light vs 4Hz stim t(72)=0.86, P=0.63; Fear+Ext Memory: no light vs 8Hz stim t(72)= 2.93, P=0.0045; no light vs 4Hz stim t(72)=3.63, P=0.0011; No Memory: no light vs 8Hz stim t(72)=0.19, P=0.96; no light vs 4Hz stim t(72)=0.24, P=0.96; 4-8-4-8 stimulation order: trial F(2, 8)=54.44, P<0.0001, stimulation F(2, 8)=1.82, P=0.22, trial x stimulation F(4, 16)=6.68, P=0.0023, n=5 mice; Holm-Sidak's multiple comparisons test; Fear Memory: no light vs. 8Hz stim t(16)=1.3, P=0.21; no light vs 4Hz stim t(16)=1.70, P=0.20; Fear+Ext Memory: no light vs 8Hz stim t(16)= 1.49, P=0.16; no light vs 4Hz stim t(16)=3.71, P=0.0038; No Memory: no light vs 8Hz stim t(16)=0.11, P=0.92; no light vs 4Hz stim t(16)=0.62, P=0.79). **(f)** Analysis of freezing scores during each stimulation interval for the 8-4-8-4 stimulation group confirms bidirectional and frequency-specific effects on post-extinction fear memory retrieval (paired two-tailed t-test: ** P<0.001; n=19 mice). **(g)** Mice without expression of ChR2 showed no significant effect of optical stimulation before or after extinction learning (two-way repeated measures ANOVA: trial F(1,10)=112.8, P<0.0001, stimulation F(2,20)=2.30, P=0.13, trial x stimulation F(2,20)=1.41, P=0.27, n=11 mice; Holm-Sidak's multiple comparisons test; Fear Memory: no light vs. 8Hz stim t(20)=0.11, P=0.91; no light vs 4Hz stim t(20)=0.89, P=0.62; Fear+Ext Memory: no light vs 8Hz stim t(20)= 1.93, P=0.13; no light vs 4Hz stim t(20)=0.86, P=0.40). **(h)** The behavioral effect of 8Hz stimulation after extinction learning is subject to a floor effect, as demonstrated by mice with lower baseline freezing having a significantly reduced effect of 8Hz stimulation compared to mice with higher baseline freezing during the post-extinction trial (left: Pearson correlation, two-tailed; Fear memory: F(1,24)=0.013, P=0.91, R^2=5.505e-04; Fear+Ext Memory: F(1,24)=12.52, P=0.0017, R^2=0.34. Right: Unpaired two-tailed t-test p=0.022, t(24)=2.45, n=26). **(i)** The lack of a 4Hz

stimulation effect prior to extinction learning cannot be explained by a behavioral ceiling effect, as demonstrated by mice with a higher baseline freezing having the same lack of effect of 4Hz stimulation as mice with a lower baseline freezing during the pre-extinction trial (left: Pearson correlation, two-tailed: Fear memory: $F(1,24)=0.052$, $P=0.82$, $R^2=0.0022$; Fear+Ext Memory: $F(1,24)=1.81$, $P=0.19$, $R^2=0.070$. Right: Mann-Whitney two-tailed test U=72.5, p=0.56, n=26). **(j)** The behavioral effect of 4Hz optical stimulation in the conditioned context (Fear+Ext) did not correlate with piriform cortex (PIR) ChR2 expression (PIR ChR2-mCherry signal normalized as a percentage of BLA ChR2-mCherry signal; data-points marked BLA-only are BLA stimulation mice analyzed in Figure 5, data-points marked as Phase are dual BLA-PFC stimulation mice in Figure 7. Non-parametric Spearman correlation: two-tailed P=0.91; r=-0.02; n=35 mice). **(k)** Comparison of behavioral effect of 4Hz stimulation in animals with either low (<20%) or high (>20%) PIR ChR2 expression (Mann-Whitney two-tailed test U=140, p=0.70, n=19 of <20%; n=16 of >20%). **(l)** The behavioral effect of 8Hz optical stimulation in the conditioned context (Fear+Ext) did not correlate with PIR ChR2 expression (non-parametric Spearman correlation: two-tailed P=0.57; r=-0.12 n=24 mice). **(m)** Comparison of behavioral effect of 8Hz stimulation in animals with either low (<20%) or high (>20%) PIR ChR2 expression (Mann-Whitney two-tailed test U=58, p=0.45, n=11 of <20%; n=13 of >20%). All error bars: mean ± SEM.

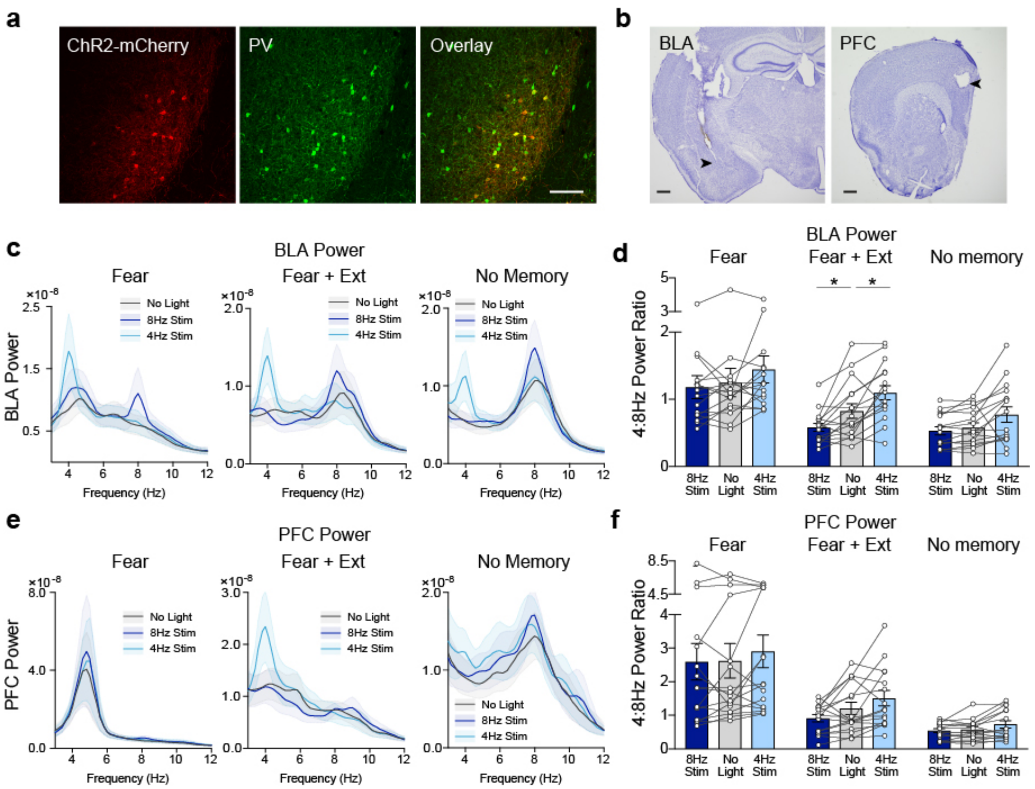

Supplementary Figure 4. Effects of BLA PV-interneuron stimulation on LFP power in the BLA and mPFC.

(a) Example confocal z-stack showing expression of ChR2-mCherry in BLA PV-interneurons. Co-localization of ChR2-mCherry with PV by immunohistochemistry was confirmed in at least the first two cohorts of mice. Scale bar=100um. **(b)** Example brightfield images of a cresyl-violet stained tissue confirming BLA and mPFC electrode locations; histological analysis was similarly conducted for all 16 mice. Scale bar: 400µm. **(c)** Averaged BLA power spectra comparing effects of 8Hz and 4Hz stimulation across trials with different memory states (n=16 mice). **(d)** 4Hz and 8Hz BLA stimulation significantly shifts BLA power but this effect is present in all trials of different memory states, as indicated by a lack of significant trial x stimulation interaction (two-way repeated measures ANOVA: trial F(2,30)=10.74, P=0.0003, stimulation F(2,30)=25.99, P<0.0001, trial x stimulation F(4,60)=1.26, P=0.30, n=16 mice; Holm-Sidak's multiple comparisons test; Fear Memory: no light vs. 8Hz stim t(60)=0.68, P=0.50; no light vs 4Hz stim t(60)=2.01, P=0.095; Fear+Ext Memory: no light vs 8Hz stim t(60)= 2.46, P=0.017; no light vs 4Hz stim t(60)=2.80, P=0.014; No Memory: no light vs 8Hz stim t(60)=0.50, P=0.62; no light vs 4Hz stim t(60)=1.97, P=0.10). **(e)** Averaged mPFC power spectra comparing effects of 8Hz and 4Hz BLA stimulation across trials with different memory states (n=16 mice). **(f)** 4Hz and 8Hz BLA stimulation shifted mPFC power but this effect is not significantly different between trials of different memory states, as indicated by a lack of significant trial x stimulation interaction (two-way repeated measures ANOVA: trial F(2,30)=14.42, P<0.0001, stimulation F(2,30)=5.94, P=0.0067, trial x stimulation F(4,60)=0.90, P=0.47, n=16 mice; Holm-Sidak's multiple comparisons test; Fear Memory: no light vs. 8Hz stim t(60)=0.19, P=0.85; no light vs 4Hz stim t(60)=1.70, P=0.18; Fear+Ext Memory: no light vs 8Hz stim t(60)=1.78, P=0.13; no light vs 4Hz

stim t(60)=1.85, P=0.13; No Memory: no light vs 8Hz stim t(60)=0.24, P=0.81; no light vs 4Hz stim t(60)=0.91, P=0.60). All error bars and shaded area: mean ± SEM

.

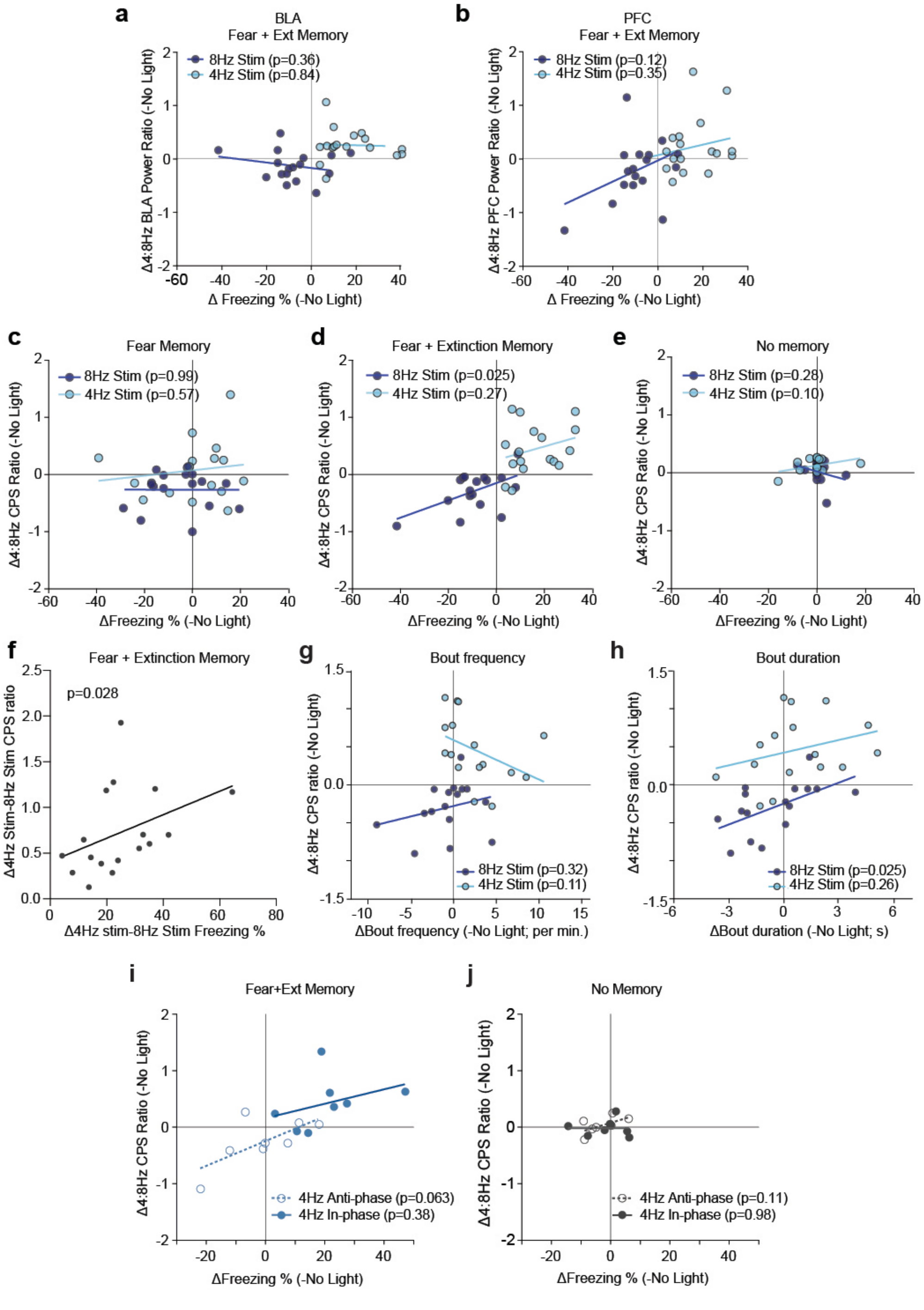

Supplementary Figure 5. Correlations between effect of stimulation on behavior and BLA and mPFC LFP measures.

(a) Correlation between stimulation-induced changes in freezing and 4:8Hz BLA power ratio in the conditioning (Fear + Extinction Memory) context (Pearson correlation, two-tailed: 8Hz stim F(1,15)=0.87, P=0.36, R^2=0.055; 4Hz stim F(1,15)=0.045, P=0.84, R^2=0.0030, n=17 mice). **(b)**

Same as in (a) but for mPFC power (Pearson correlation, two-tailed: 8Hz stim $F(1,15)=2.68$, $P=0.12$, $R^2=0.15$; 4Hz stim $F(1,15)=0.94$, $P=0.35$, $R^2=0.059$, n=17 mice). **(c)** Correlation between stimulation-induced effects on freezing and BLA-mPFC CPS in the 'Fear Memory' context (Pearson correlation, two-tailed: 8Hz stim $F(1,15)=0.00033$, $P=0.99$, $R^2=2.17e-5$; 4Hz stim $F(1,15)=0.35$, $P=0.57$, $R^2=0.022$, n=17 mice). **(d)** Same as in (c) but in the conditioned context after extinction (Fear + Extinction Memory) (Pearson correlation, two-tailed: 8Hz stim $F(1,15)=6.23$, $P=0.025$, $R^2=0.29$; 4Hz stim $F(1,15)=1.33$, $P=0.27$, $R^2=0.081$, n=17 mice). **(e)** Same as in (c-d) but in the unconditioned No Memory context (Pearson correlation, two-tailed: 8Hz stim $F(1,14)=1.28$, $P=0.28$, $R^2=0.084$; 4Hz stim $F(1,14)=3.04$, $P=0.10$, $R^2=0.18$, n=16 mice). **(f)** Correlation between difference in effect between 4Hz stim and 8Hz stim on freezing levels and on BLA-mPFC 4:8Hz CPS ratio (non-parametric Spearman correlation: two-tailed $P=0.0283$; $r=0.54$; n=17 mice). **(g)** Correlation between stimulation-induced changes in freezing bout frequency and BLA-mPFC CPS ratio (Pearson correlation, two-tailed; 8Hz Stim: $F(1,14)=1.05$, $P=0.32$, $R^2=0.070$. 4Hz Stim: $F(1,14)=2.95$, $P=0.11$, $R^2=0.17$; n=16 mice). **(h)** Same as in (g) but for bout duration (Pearson correlation, two-tailed: 8Hz Stim: $F(1,14)=6.28$, $P=0.025$, $R^2=0.31$. 4Hz Stim: $F(1,14)=1.36$, $P=0.26$, $R^2=0.089$; n=16 mice). **(i)** Correlation between 4Hz in-phase vs. 4Hz anti-phase stimulation-induced effects on freezing with BLA-mPFC CPS in the conditioned context after extinction (Fear+Extinction Memory) (Pearson correlation, two-tailed: 4Hz anti-phase $F(1,6)= 5.17$, $P=0.063$, $R^2=0.46$; 4Hz In-phase $F(1,6)=0.91$, $P=0.38$, $R^2=0.13$). **(j)** Same as in (i) but in the unconditioned (No Memory) context (Pearson correlation, two-tailed 4Hz anti-phase $F(1,6)= 3.42$, $P=0.11$, $R^2=0.36$; 4Hz In-phase $F(1,6)=0.00044$, $P=0.98$, $R^2=7.293e-05$).

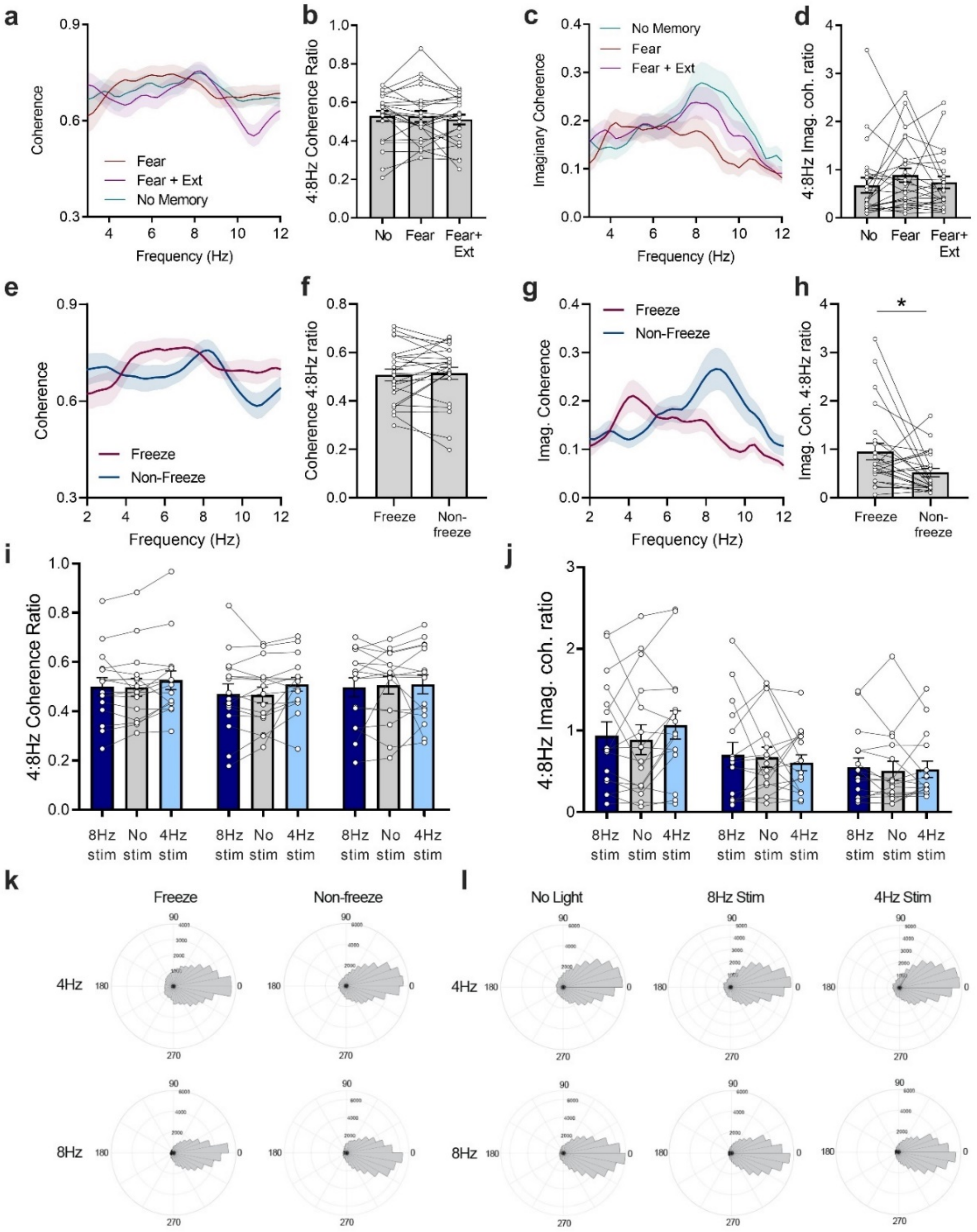

Supplementary Figure 6. Effects of BLA PV-interneuron stimulation on coherence and average phase differences across BLA-mPFC circuits.

(a-b) Fear conditioning and extinction learning have no effect on the 4:8Hz BLA-mPFC coherence ratio (a: averaged coherence spectra, n=24 mice; b: quantification as 4:8Hz coherence ratio; one-way repeated measures ANOVA: F(1.99, 45.67)=0.71, P=0.50, n=24 mice. Tukey's multiple comparisons test: No memory vs. Fear memory: P=0.99; No memory vs. Fear+Ext Memory: P=0.50; Fear vs. Fear+Ext Memory: P=0.63). **(c-d)** Same as in (a-b) but for imaginary coherence (one-way repeated measures ANOVA: F(1.71, 39.23)=1.34, P=0.27, n=24 mice. Tukey's multiple

comparisons test: No memory vs. Fear memory: P=0.35; No memory vs. Fear+Ext Memory: P=0.83; Fear vs. Fear+Ext Memory: P=0.55). **(e)** Averaged coherence spectra restricting to freezing and non-freezing periods in the Fear and No memory contexts, respectively (n=24 mice). **(f)** Quantification of coherence spectra during freeze vs. non-freeze as a 4:8Hz ratio (Wilcoxon matched-pairs test: two-tailed P=0.75, n=24 mice) **(g)** Same as in (e) but for imaginary coherence (n=24 mice). **(h)** Same as in (f) but for imaginary coherence (Wilcoxon matched-pairs test: two-tailed P=0.016, n=24 mice). **(i)** Stimulation of BLA PV-interneurons has no effect on the 4:8Hz BLA-mPFC coherence ratio (two-way repeated measures ANOVA: trial F(2,30)=0.92, P=0.41, stimulation F(2,30)=3.04, P=0.063, trial x stimulation F(4,60)=0.49, P=0.74, n=16 mice. Holm-Sidak's multiple comparisons test: Fear Memory: no light vs. 8Hz stim t(60)=0.16, P=0.88; no light vs 4Hz stim t(60)=1.43, P=0.29. Fear+Ext Memory: no light vs 8Hz stim t(60)= 0.21, P=0.83; no light vs 4Hz stim t(60)=2.07, P=0.083. No Memory: no light vs 8Hz stim t(60)=0.43, P=0.89, no light vs 4Hz stim t(60)=0.16, P=0.89). **(j)** Stimulation of BLA PV-interneurons has no effect on 4:8Hz BLA-mPFC imaginary coherence ratio (two-way repeated measures ANOVA: trial F(2,30)=6.42, P=0.0048, stimulation F(2,30)=0.29, P=0.75, trial x stimulation F(4,60)=1.15, P=0.34, n=16 mice. Holm-Sidak's multiple comparisons test: Fear Memory: no light vs. 8Hz stim t(60)=0.54, P=0.59; no light vs 4Hz stim t(60)=1.95, P=0.11. Fear+Ext Memory: no light vs 8Hz stim t(60)= 0.29, P=0.77; no light vs 4Hz stim t(60)=0.74, P=0.71. No Memory: no light vs 8Hz stim t(60)=0.50, P=0.85, no light vs 4Hz stim t(60)=0.21, P=0.85). **(k)** Circular distribution of phase differences between BLA and mPFC for 3-5Hz (4Hz) and 7-9Hz (8Hz) bandpass filtered LFPs during freezing or non-freezing periods (n=16 mice). **(l)** Same as in G but comparing stimulation periods during the post-extinction retrieval trial (n=16 mice). All error bars and shaded area: mean ± SEM.

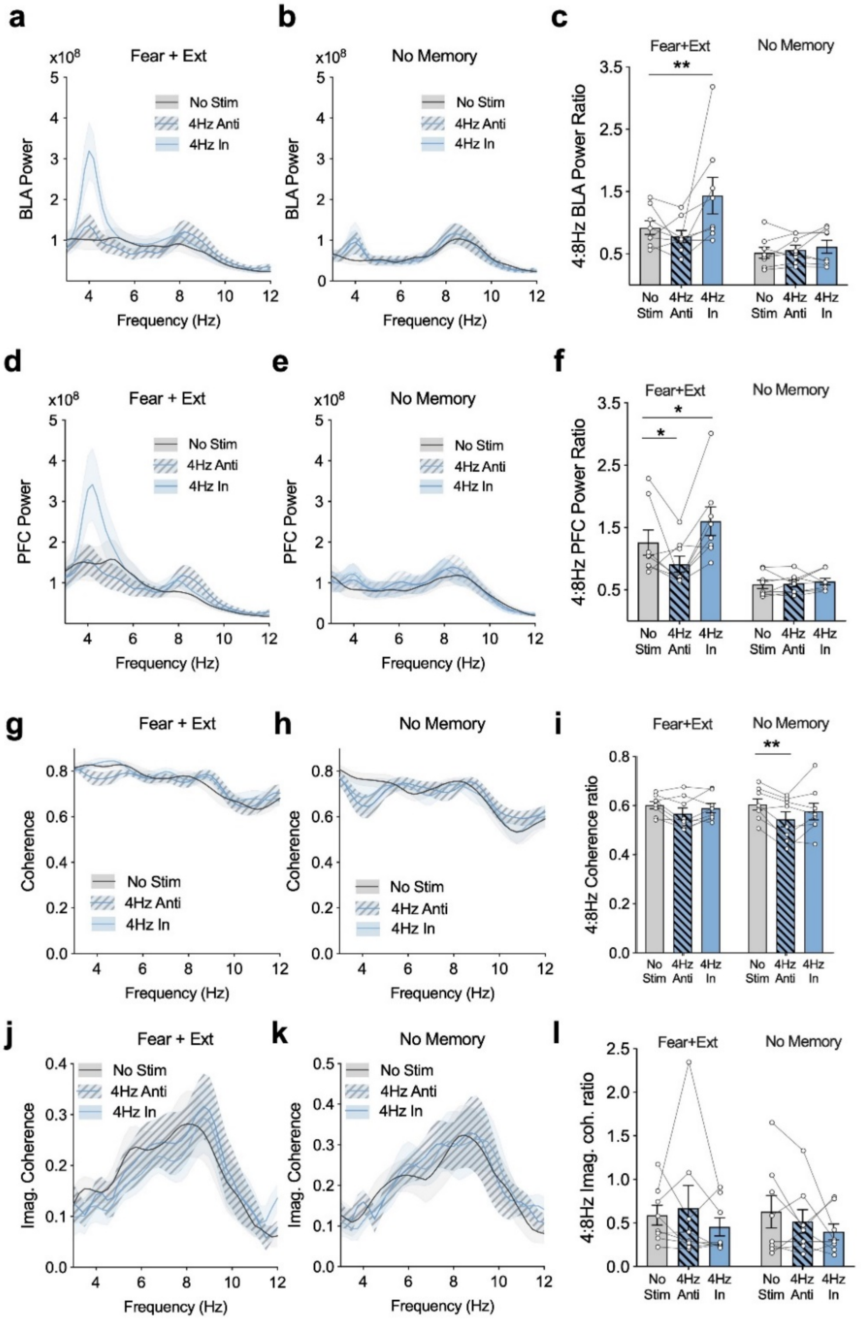

Supplementary Figure 7. Effects of in- and anti-phase 4Hz BLA-PFC stimulation on LFP power in the BLA and mPFC.

(a-b) Averaged BLA power spectra comparing no light with 4Hz anti-phase and 4Hz in-phase stimulation periods in the post-extinction conditioned context (a; fear + extinction memory state), and a novel context (b; no memory state) (n=8 mice). **(c)** 4Hz stimulation increases BLA 4:8Hz power ratio when BLA and mPFC are stimulated in-phase, but not anti-phase with one another, and only in the post-extinction conditioned context (fear + extinction memory state) (2-way RM

ANOVA: trial $F(1,7)=19.73$, $P=0.0030$, stimulation $F(2,14)=3.66$, $P=0.053$, trial x stimulation $F(2,14)=5.00$, $P=0.023$, n=8 mice. Holm-Sidak's multiple comparisons test; Fear+Ext memory state: no light vs. 4Hz anti-phase: $t(14)=1.01$, $P=0.33$; no light vs. 4Hz In-phase $t(14)=3.70$, $P=0.0048$. No memory state: no light vs. 4Hz anti-phase: $t(14)=0.34$, $P=0.75$; no light vs. 4Hz In-phase $t(14)=0.69$, $P=0.75$). **(d-e)** Same as in (a-b) but showing averaged mPFC power spectra (n=8 mice). **(f)** 4Hz in-phase stimulation increases, while 4Hz anti-phase stimulation decreases 4:8Hz mPFC power ratio in the post-extinction conditioned context (fear + extinction memory state). Neither stimulation has an effect in the novel context (no memory state). (2-way RM ANOVA: trial $F(1,7)=16.97$, $P=0.0045$, stimulation $F(2,14)=7.44$, $P=0.0063$, trial x stimulation $F(2,14)=7.32$, $P=0.0067$, n=8 mice. Holm-Sidak's multiple comparisons test; Fear+Ext memory state: no light vs. 4Hz anti-phase: $t(14)=2.85$, $P=0.026$; no light vs. 4Hz In-phase $t(14)=2.81$, $P=0.026$. No memory state: no light vs. 4Hz anti-phase: $t(14)=0.11$, $P=0.92$; no light vs. 4Hz In-phase $t(14)=0.37$, $P=0.92$). **(g-h)** Same as in (a-b) but showing averaged coherence spectra (n=8 mice). **(i)** 4Hz in-phase and anti-phase stimulation has no difference in effect on coherence across trials (2-way RM ANOVA: trial $F(1,7)=0.54$, $P=0.49$, stimulation $F(2,14)=6.33$, $P=0.011$, trial x stimulation $F(2,14)=0.87$, $P=0.44$, n=8 mice. Holm-Sidak's multiple comparisons test; Fear+Ext memory state: no light vs. 4Hz anti-phase: $t(14)=2.50$, $P=0.050$; no light vs. 4Hz In-phase $t(14)=0.90$, $P=0.38$. No memory state: no light vs. 4Hz anti-phase: $t(14)=4.34$, $P=0.0014$; no light vs. 4Hz In-phase $t(14)=2.04$, $P=0.061$). **(j-k)** Same as in (a-b) but showing averaged imaginary coherence spectra (n=8 mice). **(l)** 4Hz in-phase and anti-phase stimulation has no difference in effect on imaginary coherence across trials (2-way RM ANOVA: trial $F(1,7)=0.60$, $P=0.47$, stimulation $F(2,14)=1.21$, $P=0.33$, trial x stimulation $F(2,14)=0.60$, $P=0.56$, n=8 mice. Holm-Sidak's multiple comparisons test; Fear+Ext memory state: no light vs. 4Hz anti-phase: $t(14)=0.65$, $P=0.53$; no light vs. 4Hz In-phase $t(14)=1.080$, $P=0.51$; No memory state: no light vs. 4Hz anti-phase: $t(14)=0.90$, $P=0.38$; no light vs. 4Hz In-phase $t(14)=1.86$, $P=0.16$). All error bars and shaded area: mean ± SEM.

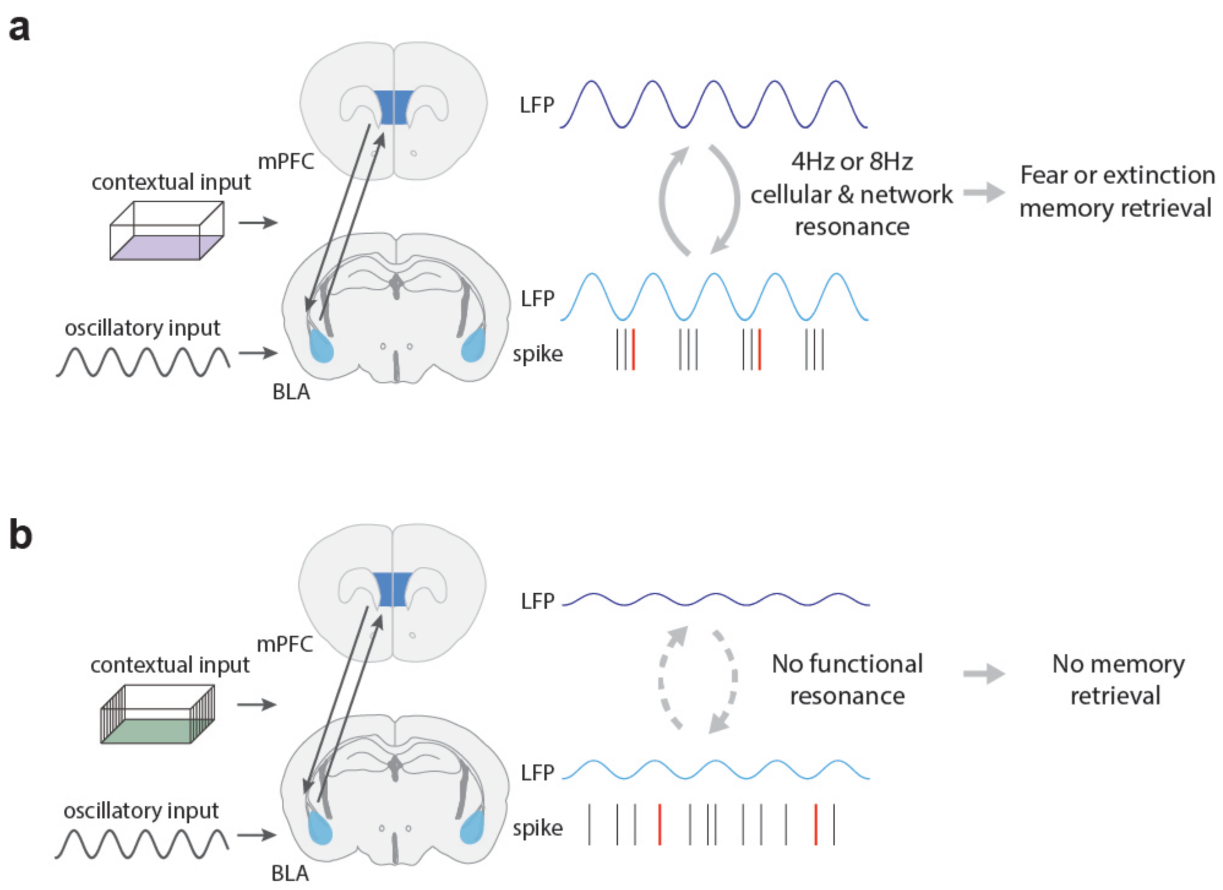

Supplementary Figure 8. Experience-dependent resonance underlies fear and extinction memory retrieval.

(a) Schematic demonstrating resonance phenomena during memory retrieval. When oscillatory input is applied to BLA in parallel with contextual input from the conditioned context, functional BLA ensembles (spiking symbolized in red) are recruited and amplification (or resonance) of the oscillatory input is observed across the BLA-mPFC network, leading to altered behavioral output. However, in **(b)** when identical oscillatory input is applied to the system in the absence of meaningful contextual input, functional ensembles are not recruited to the stimulation frequency, and network resonance is not achieved, resulting in unchanged behavioral output.
