# Supplementary material for: Experience-dependent resonance in amygdalo-cortical circuits supports fear memory retrieval following extinction
Source: Nat Commun. 2020 Aug 31;11:4358. doi: 10.1038/s41467-020-18199-w (PMC7459312; doi:10.1038/s41467-020-18199-w)
Supplement: Supplementary file 2 — Reporting Summary [file 41467_2020_18199_MOESM2_ESM.pdf]

nature research

Corresponding author(s): Leon Reijmers

Last updated by author(s): Jul 14, 2020

Reporting Summary

Nature Research wishes to improve the reproducibility of the work that we publish. This form provides structure for consistency and transparency in reporting. For further information on Nature Research policies, see our Editorial Policies and the Editorial Policy Checklist.

Statistics

For all statistical analyses, confirm that the following items are present in the figure legend, table legend, main text, or Methods section.

n/a	Confirmed	
☐	☒	The exact sample size (*n*) for each experimental group/condition, given as a discrete number and unit of measurement
☐	☒	A statement on whether measurements were taken from distinct samples or whether the same sample was measured repeatedly
☐	☒	The statistical test(s) used AND whether they are one- or two-sided<br>*Only common tests should be described solely by name; describe more complex techniques in the Methods section.*
☒	☐	A description of all covariates tested
☐	☒	A description of any assumptions or corrections, such as tests of normality and adjustment for multiple comparisons
☐	☒	A full description of the statistical parameters including central tendency (e.g. means) or other basic estimates (e.g. regression coefficient) AND variation (e.g. standard deviation) or associated estimates of uncertainty (e.g. confidence intervals)
☐	☒	For null hypothesis testing, the test statistic (e.g. *F*, *t*, *r*) with confidence intervals, effect sizes, degrees of freedom and *P* value noted<br>*Give P values as exact values whenever suitable.*
☒	☐	For Bayesian analysis, information on the choice of priors and Markov chain Monte Carlo settings
☒	☐	For hierarchical and complex designs, identification of the appropriate level for tests and full reporting of outcomes
☒	☐	Estimates of effect sizes (e.g. Cohen's *d*, Pearson's *r*), indicating how they were calculated

Our web collection on statistics for biologists contains articles on many of the points above.

Software and code

Policy information about availability of computer code

Data collection	Data was acquired using the OpenEphys system, Powerlab Labchart system (version 7; ADI instruments), Actimetrics FreezeFrame software system (version 4), Keyence system (BZ-X Analyzer 700)
Data analysis	Data was analysed using Graphpad Prism (version 7 and 8), MClust (version 4.4), as well as functions available in the Fieldtrip (http://www.fieldtriptoolbox.org/) and EEGLAB (https://sccn.ucsd.edu/eeglab) toolboxes.

For manuscripts utilizing custom algorithms or software that are central to the research but not yet described in published literature, software must be made available to editors and reviewers. We strongly encourage code deposition in a community repository (e.g. GitHub). See the Nature Research guidelines for submitting code & software for further information.

Data

Policy information about availability of data

All manuscripts must include a data availability statement. This statement should provide the following information, where applicable:

- Accession codes, unique identifiers, or web links for publicly available datasets
- A list of figures that have associated raw data
- A description of any restrictions on data availability

Source data are provided with this paper. This statement is included in the manuscript.

[illegible]

[illegible]

- [x] [illegible]
- [ ] [illegible]
- [ ] [illegible]

[illegible]

[illegible]

[illegible]

[illegible]	[illegible]
[illegible]	[illegible]
[illegible]	[illegible]
[illegible]	[illegible]
[illegible]	[illegible]

[illegible]

[illegible]

[illegible]

[illegible]	[illegible]	
[ ]	[x]	[illegible]
[x]	[ ]	[illegible]
[x]	[ ]	[illegible]
[ ]	[x]	[illegible]
[ ]	[x]	[illegible]
[x]	[ ]	[illegible]
[x]	[ ]	[illegible]

[illegible]

[illegible]	[illegible]	
[x]	[ ]	[illegible]
[x]	[ ]	[illegible]
[x]	[ ]	[illegible]

[illegible]

[illegible]	[illegible]
[illegible]	[illegible]

[illegible]

[illegible]

[illegible]	[illegible]
[illegible]	[illegible]
[illegible]	[illegible]

Ethics oversight	All animal procedures were performed in accordance with the NIH Health Guide for the Care and Use of Laboratory Animals and were approved by the Tufts University Institutional Animal Care and Use Committee.

Note that full information on the approval of the study protocol must also be provided in the manuscript.

Human research participants

Policy information about studies involving human research participants

Population characteristics	n/a
Recruitment	n/a
Ethics oversight	n/a

Note that full information on the approval of the study protocol must also be provided in the manuscript.
